# A receptor required for chitin perception facilitates arbuscular mycorrhizal associations and distinguishes root symbiosis from immunity

**DOI:** 10.1016/j.cub.2024.03.015

**Published:** 2024-04-22

**Authors:** Jingyi Zhang, Jongho Sun, Chai Hao Chiu, David Landry, Kangping Li, Jiangqi Wen, Kirankumar S. Mysore, Sébastien Fort, Benoit Lefebvre, Giles E.D. Oldroyd, Feng Feng

**Affiliations:** 1 Department of Biochemistry and Molecular Biology, Oklahoma State University, Stillwater, OK 74078, USA; 2 Crop Science Centre, Department of Plant Sciences, University of Cambridge, Cambridge CB3 0LE, UK; 3 Laboratory of Plant-Microbe-Environment Interactions (LIPME), Université de Toulouse, INRAE, CNRS, Castanet-Tolosan 31326, France; 4 Institute for Agricultural Biosciences, Oklahoma State University, Ardmore, OK 73401, USA; 5 Department of Plant and Soil Sciences, Oklahoma State University, Stillwater, OK 74078, USA; 6 Université de Grenoble Alpes, CNRS, CERMAV, 38000 Grenoble, France

**Keywords:** *Medicago truncatula*, arbuscular mycorrhizal fungi, chitooligosaccharides, lysin motif receptor-like kinase, plant immunity, plant symbiosis

## Abstract

Plants establish symbiotic associations with arbuscular mycorrhizal fungi (AMF) to facilitate nutrient uptake, particularly in nutrient-limited conditions. This partnership is rooted in the plant’s ability to recognize fungal signaling molecules, such as chitooligosaccharides (chitin) and lipo-chitooligosaccharides. In the legume *Medicago truncatula*, chitooligosaccharides trigger both symbiotic and immune responses via the same lysin-motif-receptor-like kinases (LysM-RLKs), notably CERK1 and LYR4. The nature of plant-fungal engagement is opposite according to the outcomes of immunity or symbiosis signaling, and as such, discrimination is necessary, which is challenged by the dual roles of *CERK1*/*LYR4* in both processes. Here, we describe a LysM-RLK, *LYK8*, that is functionally redundant with *CERK1* for mycorrhizal colonization but is not involved in chitooligosaccharides-induced immunity. Genetic mutation of both *LYK8* and *CERK1* blocks chitooligosaccharides-triggered symbiosis signaling, as well as mycorrhizal colonization, but shows no further impact on immunity signaling triggered by chitooligosaccharides, compared with the mutation of *CERK1* alone. LYK8 interacts with CERK1 and forms a receptor complex that appears essential for chitooligosaccharides activation of symbiosis signaling, with the *lyk8*/*cerk1* double mutant recapitulating the impact of mutations in the symbiosis signaling pathway. We conclude that this novel receptor complex allows chitooligosaccharides activation specifically of symbiosis signaling and helps the plant to differentiate between activation of these opposing signaling processes.

## Highlights

• *Medicago LYK8* is functionally redundant with *CERK1* for mycorrhizal fungi symbiosis
• LYK8 cannot bind to chitin but is involved in chitin-mediated symbiosis signaling
• LYK8 is not required for chitin-induced plant immunity
• LYK8 forms a receptor complex with CERK1 and DMI2 to activate symbiosis signaling

## Graphical abstract

**Figure:**
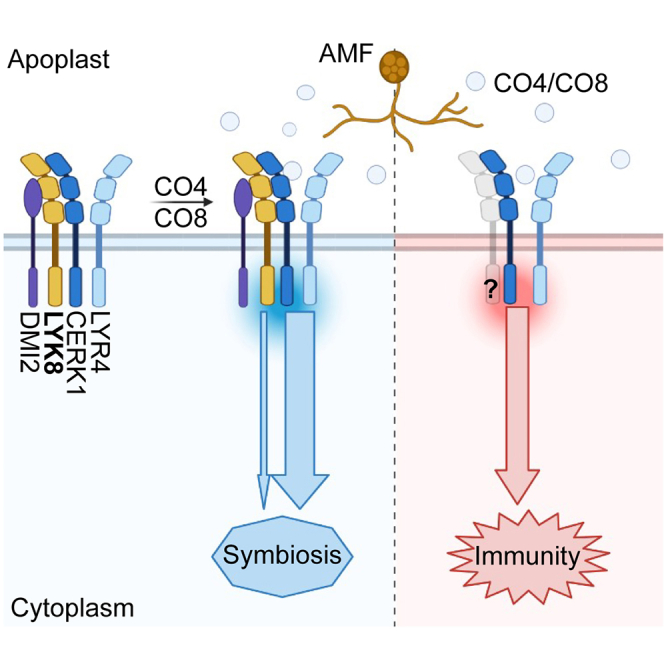

## Teaser

The establishment of arbuscular mycorrhizal fungi (AMF) symbiosis necessitates the recognition of fungal signals by plant receptors. Zhang et al. discover a LysM receptor that is functionally redundant with CERK1 for perceiving chitin produced by AMF, thereby specifically activating symbiosis signaling and enhancing AMF colonization.

## Introduction

The majority of terrestrial plant species can establish a symbiotic relationship with arbuscular mycorrhizal fungi (AMF) to enhance nutrient uptake.^1^^,^^2^ This symbiosis boosts plant growth and plays a pivotal role in the regulation of the global terrestrial biogeochemical cycles.^3^ For this relationship to be established, plants must recognize AMF through the activation of a conserved symbiosis signaling pathway.^1^^,^^4^ This activation occurs through the perception of fungal signaling molecules, known as “Myc Factors.” ^5^ Myc factors comprise chitooligosaccharides (Myc-COs) from fungal cell walls,^6^^,^^7^ which are oligomers of *N*-acetyl glucosamine, and lipo-chitooligosaccharides (Myc-LCOs) secreted by AMF.^8^ Myc-LCO is distinguished by substitutions on the nonreducing end of the molecule of *N*-acyl moieties and by additional decorations onto this basic backbone.^8^ These LCOs are structurally similar to the nodulation factors (NFs) generated by symbiotic nitrogen-fixing rhizobium bacteria.^8^ Upon CO/LCO perception, symbiosis signaling is activated through nuclear calcium oscillations and resultant symbiotic gene expression to facilitate AMF infection.^4^ Additionally, co-inoculation of CO and AMF promotes strigolactone biosynthesis, stimulating intracellular accommodation and arbuscule development of AMF in plant roots.^9^

Studies in various plant species have demonstrated that plant lysin-motif receptor-like kinases (LysM-RLKs) play a central role in recognizing CO and LCO molecules.^10^^,^^11^^,^^12^^,^^13^^,^^14^ In the legume *Medicago truncatula* (*M. truncatula*), CO oligomers ranging from 4 to 8 units can activate symbiosis signaling.^6^^,^^7^^,^^15^ Receptors required for this process include two LysM-RLKs, *MtCERK1* (hereafter designated as *CERK1*) and *MtLYR4* (hereafter *LYR4*).^7^^,^^16^ Both receptors function in the CO-activation of nuclear calcium oscillations and resultant transcriptional upregulation of symbiotic gene expression, upon treatments with either CO4 (*N*-acetyl chitotetraose) or CO8 (*N*-acetyl chitooctaose).^7^ *cerk1* displays a significant reduction in AMF colonization, highlighting a role for CO signaling in arbuscular mycorrhizal symbiosis (AMS).^7^^,^^16^ This has been similarly observed in other species where *CERK1* orthologs have been mutated, such as rice (*Oryza sativa*), pea (*Pisum sativum*), banana (*Musa spp.*), and *Parasponia andersonii* (*P. andersonii*).^17^^,^^18^^,^^19^^,^^20^^,^^21^^,^^22^^,^^23^ Besides CO, AMF also produce LCO,^8^ and several LysM-RLKs have been shown to bind LCO in legumes^24^^,^^25^^,^^26^^,^^27^^,^^28^^,^^29^^,^^30^ and act as pivotal components for LCO detection in the root nodule symbiosis (RNS), notably *MtNFP*/*LjNFR5* and *MtLYK3*/*LjNFR1* in legumes *M. truncatula* and *Lotus japonicus*.^31^^,^^32^^,^^33^^,^^34^^,^^35^^,^^36^^,^^37^ These LCO receptors, which are essential for the RNS, also contribute to the AMS, as evidenced by a quantitative reduction in AMF colonization in the *cerk1*/*nfp* double mutant, as compared with *cerk1* alone.^7^ Furthermore, *NFP* orthologs in tomato (*Solanum lycopersicum*), *Petunia hybrida*, and barley (*Hordeum vulgare*) contribute to AMF colonization,^38^^,^^39^^,^^40^^,^^41^ although, at least in barley, the function of *NFP* homologs is not limited to LCO signaling alone, as they also contribute to CO signaling.^41^ Unlike the case for the RNS, for AMF infection, no mutation in a single LysM-RLK, in any species, leads to almost complete abolishment of AMF colonization,^7^^,^^29^^,^^42^^,^^43^ suggesting the presence of additional unknown receptors involved in AMF induction of symbiosis signaling.

Besides their role as symbiotic signals, COs also act as microbe-associated molecular patterns (MAMPs), triggering plant immune responses, such as the generation of reactive oxygen species (ROS), mitogen-activated protein kinase (MAPK) phosphorylation, and initiation of defense gene expression.^2^^,^^14^^,^^44^^,^^45^ Recognition of CO8, and to a lesser extent CO4, activates defense responses in various plant species, and this requires *CERK1* and *LYR4* in *M. truncatula*.^7^^,^^45^ Activation of both immunity and symbiosis signaling through the same receptors^7^^,^^16^^,^^18^^,^^19^^,^^20^^,^^21^ suggests that plants might not be able to differentiate AMF from pathogenic fungi, solely through CO recognition.^7^ To establish a successful infection in plant roots, AMF can suppress plant immunity through LCO, short-chain CO, or secreted effectors and proteins.^7^^,^^40^^,^^46^^,^^47^^,^^48^^,^^49^ However, how plants discriminate between the activation of immunity and symbiosis signaling remains unknown.

*CERK1* is classified within the LYK subfamily of LysM-RLKs with potentially active kinase domains.^11^ This LYK family has 11 members (*MtLYK1*–*MtLYK11*; *CERK1* is *MtLYK9*)^11^ in *M. truncatula*, which is greater than the equivalent in the non-symbiotic plant species *Arabidopsis thaliana* (*A. thaliana*). This expansion may suggest an evolutionary adaptation for engaging in symbiotic microbe associations. In *M. truncatula*, *CERK1* is the major receptor mediating AMF recognition and symbiosis, but its mutant phenotype suggests functional redundancy with other receptors that still allow some level of AMF colonization.^7^^,^^16^ In this study, we identified a further LysM-RLK in the *CERK1* family, *MtLYK8* (*LYK8* hereafter), which exhibits functional redundancy with *CERK1* in the AMS but plays no role in CO-activated plant immunity. We demonstrate the dominance of CO signaling in AMS and show how *LYK8* differentiates between CO-activation of symbiosis and immunity signaling.

## Results

### *LYK8* is functionally redundant with *CERK1* for AMF colonization

**Figure 1:**
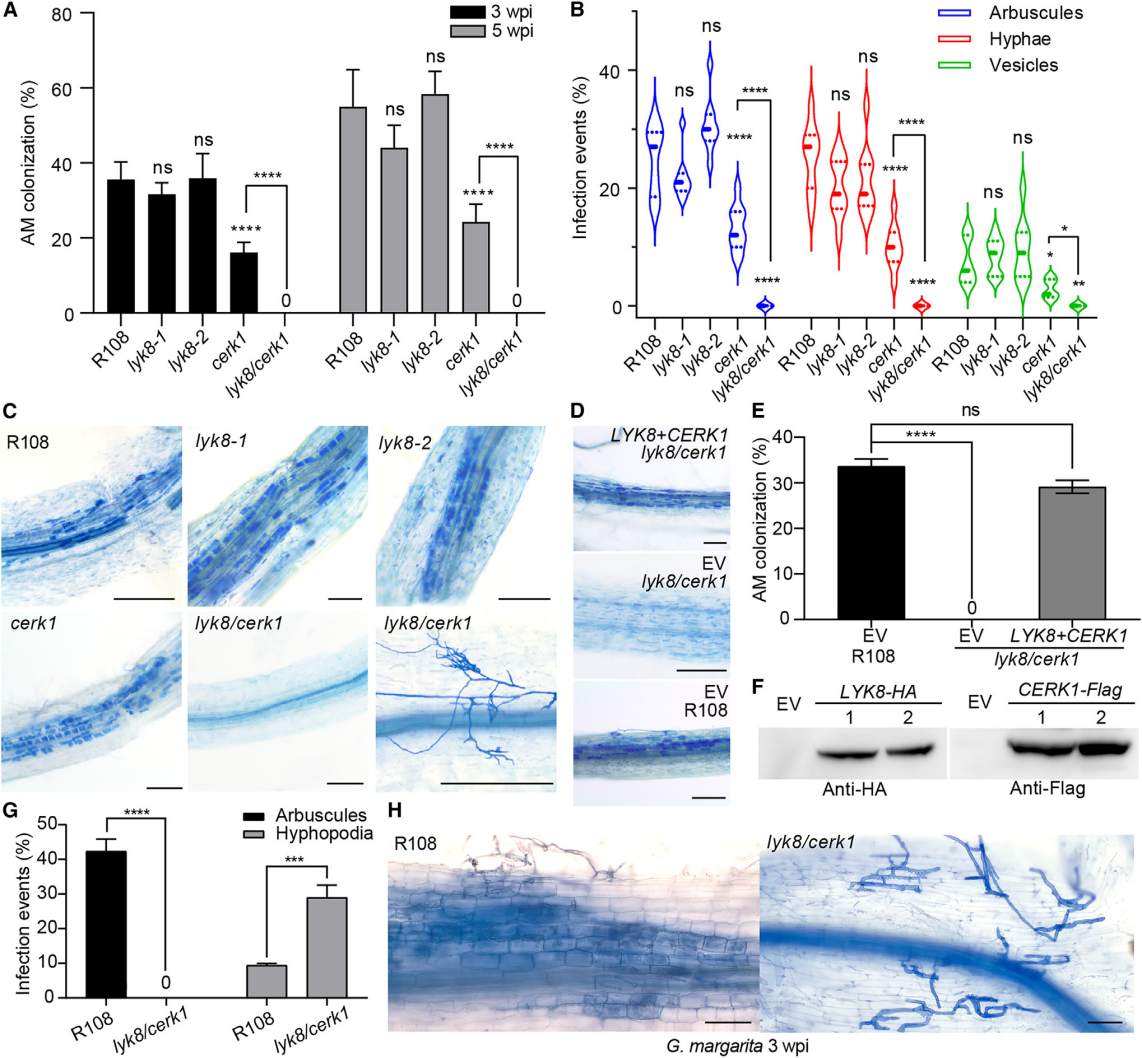
*lyk8/cerk1* double mutant exhibited no AMF colonization (A) Colonization of *R. irregularis* is represented as the percentage of root length colonization, performed at 3 and 5 weeks post inoculation (wpi). (B) Violin plot illustrates infection events quantified at 3 wpi. This experiment was repeated three times with similar results. (C) Images of *R. irregulars* inoculated roots stained with ink. The hyphopodia (bottom right image) was observed in the roots of 5 wpi of high concentration of fungal spores. Scale bars, 150 μm. (D and E) AMF colonization and corresponding images from a complementation experiment in *lyk8/cerk1* double mutant by hairy root transformation. The empty vector (EV) was transferred as a control. Scale bars, 150 μm. (F) The expression levels of *LYK8* and *CERK1* in the complementation roots were detected by western blot. Results from two individual transgenic roots are shown. (G) Arbuscules and hyphopodia were quantified in plants inoculated with *G. margarita* at 3 wpi. Statistically significant differences were determined using Student’s t test (mean ± SEM, n = 10). (H) Images show the ink-stained colonization of *G. margarita* in plant roots. Scale bars, 100 μm. (A, B, and E) Asterisks denote statistical significance as calculated by one-way ANOVA and Tukey’s multiple comparison (mean ± SEM, n = 10). See also Figures S1–S3.

To uncover LysM-RLKs in the LYK family that might participate in AMS, we obtained *Tnt1* insertion mutant lines for several LysM-RLK genes,^11^^,^^50^ including *MtLYK6*, *MtLYK7*, *MtLYK8*, and *MtLYK10* in *M. truncatula*. The homozygous mutant alleles were validated for the absence of detectable transcripts using semi-quantitative reverse transcription polymerase chain reaction, and they were designated as *lyk6*, *lyk7*, *lyk8* (*lyk8-1* and *lyk8-2*; two mutant alleles with insertions in different domains of LYK8), and *lyk10*, respectively (Figures S1A–S1C). Subsequently, wild-type and mutant plant roots were inoculated with *Rhizophagus irregularis* (*R. irregularis*) spores to assess fungal colonization. As a control, the *cerk1* mutant displayed significantly reduced fungal infection and colonization, consistent with prior observations^7^^,^^16^ (Figures 1A, 1B, S1D, and S1E). The mutations of *LYK6*, *LYK7*, *LYK8*, and *LYK10*, individually, appeared to have no effect on total AMF colonization and fungal infection (Figures 1A, 1B, S1D, and S1E). To investigate whether these LysM-RLKs function redundantly with *CERK1* for AMS, we genetically crossed *cerk1* with the new receptor mutants, producing several double-mutant lines. Surprisingly, when inoculated with a low concentration of spores, *lyk8-1/cerk1* (*lyk8/cerk1* hereafter) roots exhibited a complete loss of arbuscules, intercellular hyphae, and vesicles, while infection events in *lyk6/cerk1*, *lyk7/cerk1*, and *lyk10/cerk1* were comparable to those in *cerk1* alone (Figures 1A–1C, S1D, and S1E). Higher doses of AMF inoculum led to higher levels of hyphopodium development on the root surface of *lyk8/cerk1* than on the root surface of wild-type plants, but the fungal hyphae did not penetrate the rhizodermis to form arbuscules in the mutant (Figures 1C and S2A). This deficiency in fungal colonization persisted, even extending to 7 weeks post inoculation (Figure S2C). Additionally, co-cultivation of wild-type nurse plants with *lyk8/cerk1* increased the number of hyphopodia development in the mutant but failed to restore AMF infection and arbuscule development (Figures S2A and 2B). Altogether, this demonstrates the persistent lack of fungal infection, even with increased inoculum strength and extended inoculation time.

To confirm that the mutation of *LYK8* in the *cerk1* background hinders fungal colonization, we undertook a genetic complementation of the *lyk8/cerk1* mutant using the coding sequences of *LYK8* and *CERK1*. We introduced both genes in a single construct, driven by their native promoters, into *lyk8/cerk1* roots via *Agrobacterium rhizogenes*-mediated hairy root transformation. Successful transformation resulted in complete restoration of fungal infection and colonization (Figures 1D and 1E), accompanied by 100% complementary events in ten individual transgenic roots in which the expression of LYK8 and CERK1 proteins were detected in the roots (Figure 1F). These data reveal that *LYK8* is redundant with *CERK1* in AMS. In the single *lyk8* mutant, the signaling function is fulfilled by the major receptor *CERK1*, resulting in fungal colonization similar to that of the wild-type plant (Figures 1A–1C).

To determine whether the *lyk8/cerk1* mutant is specifically deficient in symbiosis with *R. irregularis* or impacts root symbiosis with other AMF as well, we inoculated wild-type and *lyk8/cerk1* mutant plants with *Gigaspora margarita* (*G. margarita*), which is phylogenetically distant from the model *R. irregularis*. 3 weeks post inoculation, only hyphopodia were observed, with a complete absence of arbuscules in the cortical cells of the *lyk8/cerk1* mutant (Figures 1G and 1H). This finding further supports the critical role of *LYK8* and *CERK1* in facilitating symbiotic relationships between plant roots and various AMF species.

AMS and RNS share the common symbiosis signaling pathway.^4^ To examine whether *LYK8* and *CERK1* play a role in the recognition of rhizobia, we inoculated *Ensifer meliloti Em*1021 on wild-type and mutant roots. We observed that mutation neither in *LYK8* alone nor in conjunction with *CERK1* impaired nodulation, suggesting their specific relevance in the AMS (Figure S2D).

Studies have shown that AMF can induce lateral root primordia formation and increase lateral root densities in a *CERK1*-dependent manner in diverse plant species, including *M. truncatula*.^51^ To investigate whether *LYK8* also plays a role in root development in response to AMF, we assessed the root architecture response of wild-type and receptor mutant plants. Interestingly, both *lyk8* and *cerk1* plants exhibited an increase in first-order lateral root numbers in the absence of AMF inoculation, suggesting their potential involvement in lateral root development (Figure S2G). However, *LYK8* appears to have no role in the regulation of primary root growth (Figure S2H). Notably, the promotion of lateral root number by AMF was absent in *lyk8* plants, similar to the observations in *cerk1* and *lyk8/cerk1* mutants (Figure S2G), highlighting that *LYK8* and *CERK1* are both essential for AMF-induced root responses and do not play redundant roles. This indicates that these two receptors might form a receptor complex for the perception of AMF-produced signal molecules, thereby regulating root development.

To understand how *CERK1* and *LYK8* occupy overlapping signaling functions in the AMS, we examined their promoter activity during AMF infection. Both *LYK8* and *CERK1* exhibited similar expression patterns in root tissues, as evidenced by promoter β-glucuronidase (GUS) assays and wheat germ agglutinin (WGA) staining (Figures S3A–S3H). Without *R. irregularis* inoculation, the promoters of both *LYK8* and *CERK1* were expressed throughout various root cell layers (Figures S3A and S3B). These expression patterns were maintained during the early stages of AMF infection, including hyphopodium formation (Figures S3C and S3D) and the extension of intraradical hyphae into the root cells (Figures S3E and S3F). Interestingly, fungal colonization slightly increased receptor expression in arbuscule-containing cells (Figures S3G and S3H), consistent with the observations found in the *Medicago* gene expression database,^47^ suggesting a modality of positive feedback colonization in root cells and receptor expression. However, neither *LYK8* nor *CERK1* alone are required for arbuscule development^7^ (Figure S1F). Quantitative real-time polymerase chain reaction (real-time qPCR) revealed an induction of *LYK8* expression post fungal root colonization (Figure S4A) that is likely the result of *LYK8* transcriptional induction by CO and LCO (Figure S4B). Taken together, these findings demonstrate that *LYK8* functions synergistically with *CERK1* in AMF colonization and that these two receptors show very similar expression patterns.

### LYK8 cannot bind to CO but is involved in CO-medicated symbiosis signaling

**Figure 2:**
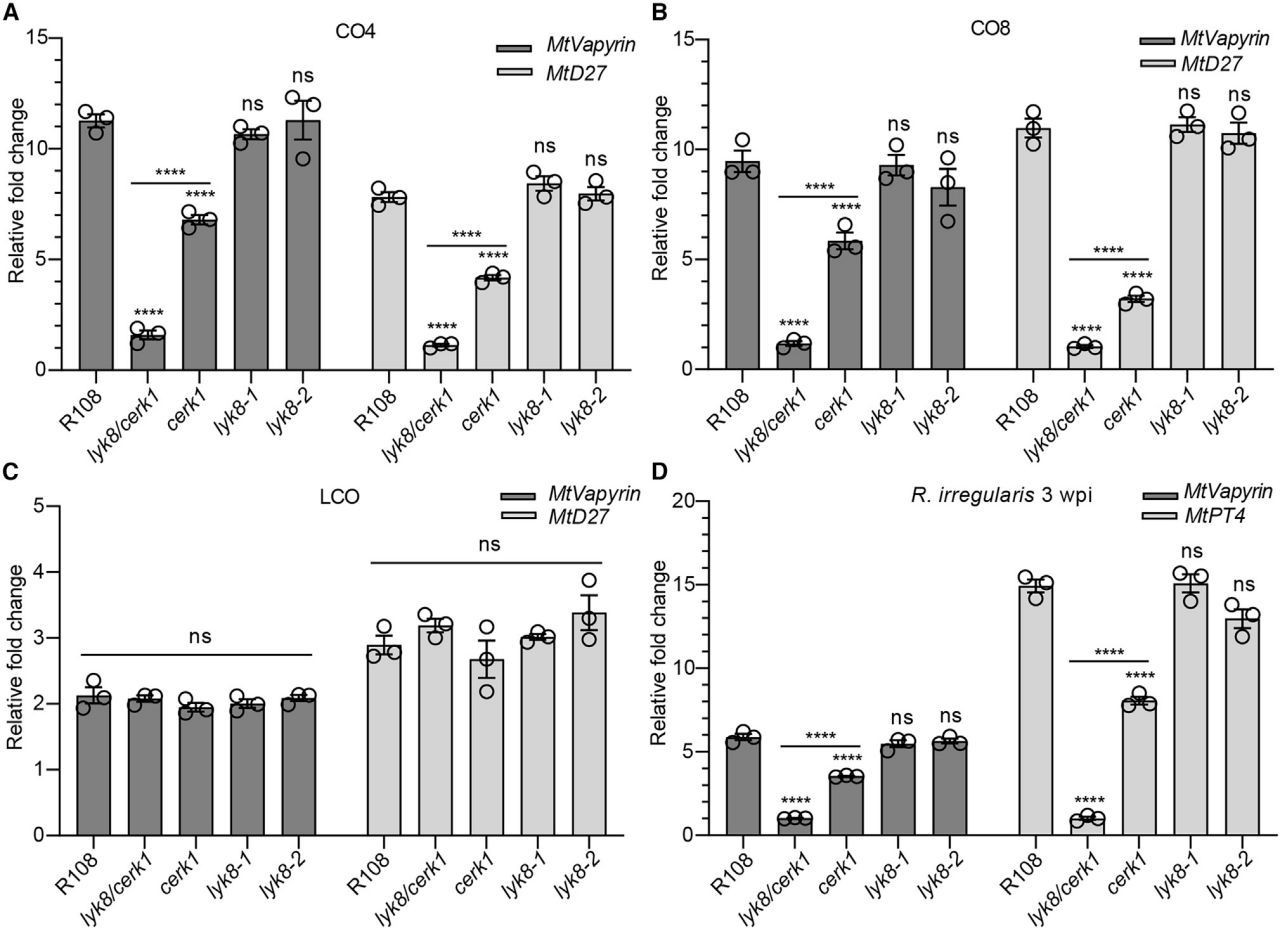
*lyk8/cerk1* completely interrupted the expression of symbiotic gene induced by CO and AMF (A–C) Real-time qPCR analysis assessed the expression of symbiotic marker genes in the roots of both the wild type and receptor mutants following treatments with H_2_O, 10^−8^ M CO4 (A), 10^−8^ M CO8 (B), and 10^−8^ M LCO (C) for 6 h. Relative fold change compared with individual water treatments is shown. (D) The relative fold change represents the expression of *MtVapyrin* and *MtPT4* detected in the plant roots 3 wpi with *R. irregularis*, compared with the expression in non-inoculated roots. (A–D) Asterisks denote statistical significance as calculated by one-way ANOVA and Tukey’s multiple comparison (mean ± SEM, n = 8). These results have three independent biological replications. See also Figure S4.

The essential role of *LYK8* and *CERK1* in fungal infection implies that similar to *CERK1*, *LYK8* may participate in early symbiosis signaling initiated by symbiotic elicitors. To test this, we evaluated CO- and LCO-triggered gene expression of symbiotic marker genes^7^ in *lyk8-1*, *lyk8-2*, *cerk1*, and *lyk8/cerk1* mutants. We found that wild-type plants exhibited CO4- and CO8-driven induction of symbiotic genes, with this induction being partially dependent on *CERK1*, but independent of *LYK8* (Figures 2A, 2B, S4C, and S4D). However, the *lyk8/cerk1* showed no detectable induction of the tested genes by CO, while maintaining their induction by LCO (Figures 2A–2C, S4C, and S4D). During *R. irregularis* inoculation, the induction of *MtVapyrin*, *MtPT4*, and *MtLYK10* was disrupted in *lyk8/cerk1* roots after 3 and 5 weeks of inoculation, in line with the colonization of this mutant (Figures 2D, S4E, and S4F). Our previous studies highlighted the combined importance of CO and LCO signaling for AMS, noting a significant reduction of AMF colonization in *cerk1/nfp* roots compared with *cerk1* alone.^7^ Interestingly, AMF colonization and infection patterns showed no defects in *lyk8/nfp* roots, indicating the dominant role of *CERK1* in CO signaling that might compensate for the deficiency of *lyk8/nfp* in AMF colonization (Figures S2E and S2F). Collectively, our findings demonstrate that *LYK8* and *CERK1* have overlapping roles in modulating CO-triggered symbiotic gene expression.

**Figure 3:**
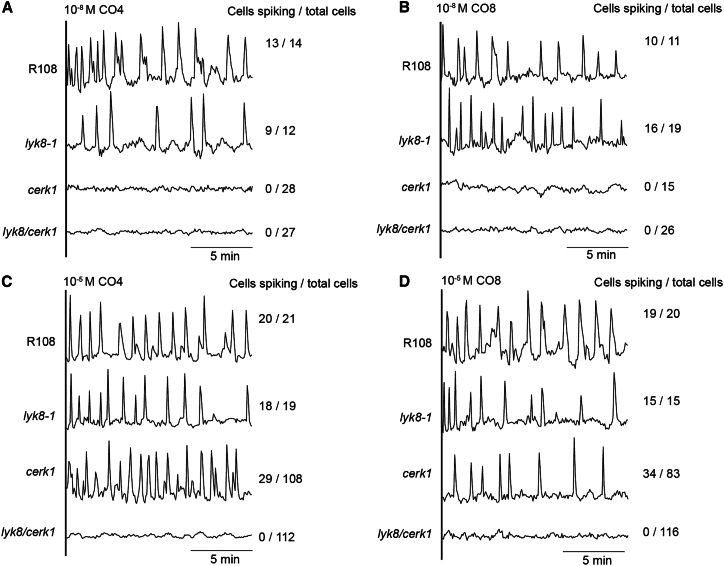
CO-triggered calcium oscillations are abolished in *lyk8/cerk1* (A–D) Representative calcium traces of *M. truncatula* atrichoblasts from lateral roots were recorded in wild type, *lyk8-1*, *cerk1*, and *lyk8/cerk1* in response to both a low concentration (10^−8^ M) of CO4 (A) and CO8 (B), as well as a high concentration (10^−5^ M) of CO4 (C) and CO8 (D). The traces denote the ratio of yellow fluorescent protein (YFP) to cyan fluorescent protein (CFP) in arbitrary units. The numbers marked on the right of the traces indicate the number of cells responding compared with the total number of cells analyzed. See also Figure S5.

Periodic calcium oscillations in the nucleus serve as an indicator of symbiosis signaling, activated by both CO and LCO.^15^ To delve deeper into the role of *LYK8* in CO-triggered symbiosis signaling, we analyzed calcium oscillations in the roots of wild type and receptor mutants using the calcium reporter, Yellow Cameleon YC3.6.^7^ In line with prior research, the *cerk1* single mutant displayed a disruption in calcium oscillations in atrichioblast cells when exposed to low concentrations of CO4 and CO8^7^ (10^−8^ M; Figures 3A and 3B). However, *cerk1* still showed a response when exposed to high concentrations of these molecules (10^−5^ M; Figures 3C and 3D), suggesting the involvement of additional receptor(s) at these concentrations of CO. In contrast to *CERK1*, the mutation of *LYK8* alone exhibited no defect in CO4- and CO8-induced nuclear calcium oscillations (Figures 3A–3D). In the *cerk1/lyk8* double mutant, we observed a complete loss of CO-induced calcium response, even at exceptionally high concentrations of CO4 and CO8 (10^−4^ M; Figures 3C, 3D, and S5). This observation underscores the redundant role of *LYK8* and *CERK1* in CO signaling. The LCO-triggered calcium response in *lyk8* and *lyk8/cerk1* mutants mirrored that of wild-type plants (Figure S5). These data, combined with the gene expression and root nodulation findings (Figures 2C and S2D), indicate that *LYK8* plays a specific role in CO, but not LCO, signaling in *M. truncatula*.

**Figure 4:**
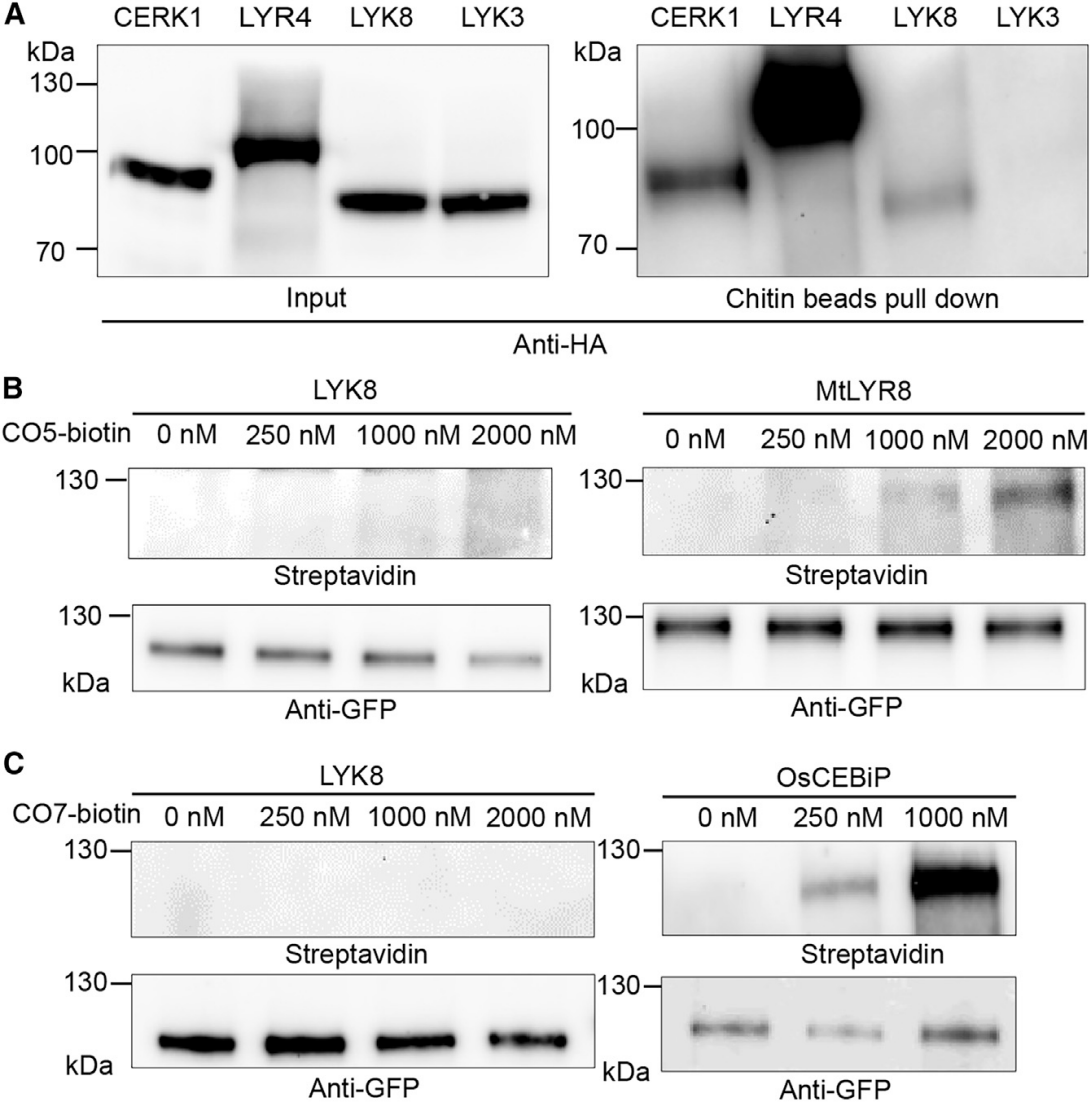
LYK8 protein weakly binds to chitin beads but not to CO5 and CO7 (A) The LYK8 protein expressed in *N. benthamiana* leaves exhibits weak binding to chitin resin beads. CERK1 and LYR4 are positive controls. LYK3, the LCO receptor, is the negative control. (B and C) Detecting the binding affinity of the LYK8 protein with CO5 and CO7. Chimeric YFP-tagged proteins were expressed in *N. benthamiana* leaves followed by incubation with CO5-biotin or CO7-biotin. The proteins were enriched using anti-GFP beads, and their presence was confirmed via western blotting with anti-GFP antibody and streptavidin. MtLYR8 served as the positive control for CO5 binding (B), and OsCEBiP was the positive control for CO7 binding (C).

To determine whether LYK8 functions as a CO receptor, we transiently expressed LYK8 in *Nicotiana benthamiana* (*N. benthamiana*) leaves and assessed its ability to bind to chitin resin beads through a pull-down assay. The previously reported CO receptors, CERK1 and LYR4, served as positive controls, while the LCO receptor LYK3 served as the negative control.^33^^,^^36^ The result demonstrated that both LYR4 and CERK1 possess a strong affinity for chitin beads, consistent with previous studies^7^ (Figure 4A). LYK8 displayed much weaker chitin binding relative to CERK1 and LYR4, while no binding was evident for LYK3 (Figure 4A). However, the weak binding observed was unable to withstand the stringent washes following the chitin bead pull-down assay (data not shown). Additionally, we employed cross-linkable biotinylated versions of CO5 (CO5-biotin) and CO7 (CO7-biotin) to evaluate LYK8’s binding affinity to COs. Consistent with the results from the chitin beads, neither CO5-biotin nor CO7-biotin was able to bind to LYK8 at any concentration (Figures 4B and 4C). In contrast, as positive controls, the *M. truncatula* MtLYR8 protein demonstrated binding ability to CO5-biotin,^52^ and the rice OsCEBiP exhibited high-affinity binding ability to CO7-biotin^53^ (Figures 4B and 4C). These data suggest that LYK8 might not be sufficient to bind with CO and is likely to function as a co-receptor for LYK9 and LYR4 in the perception of CO molecules. Nevertheless, we cannot exclude the possibility of a weak binding affinity of LYK8 for COs, undetectable in the assays that we performed.

### LYK8 appears to have no role in CO-triggered plant immunity

**Figure 5:**
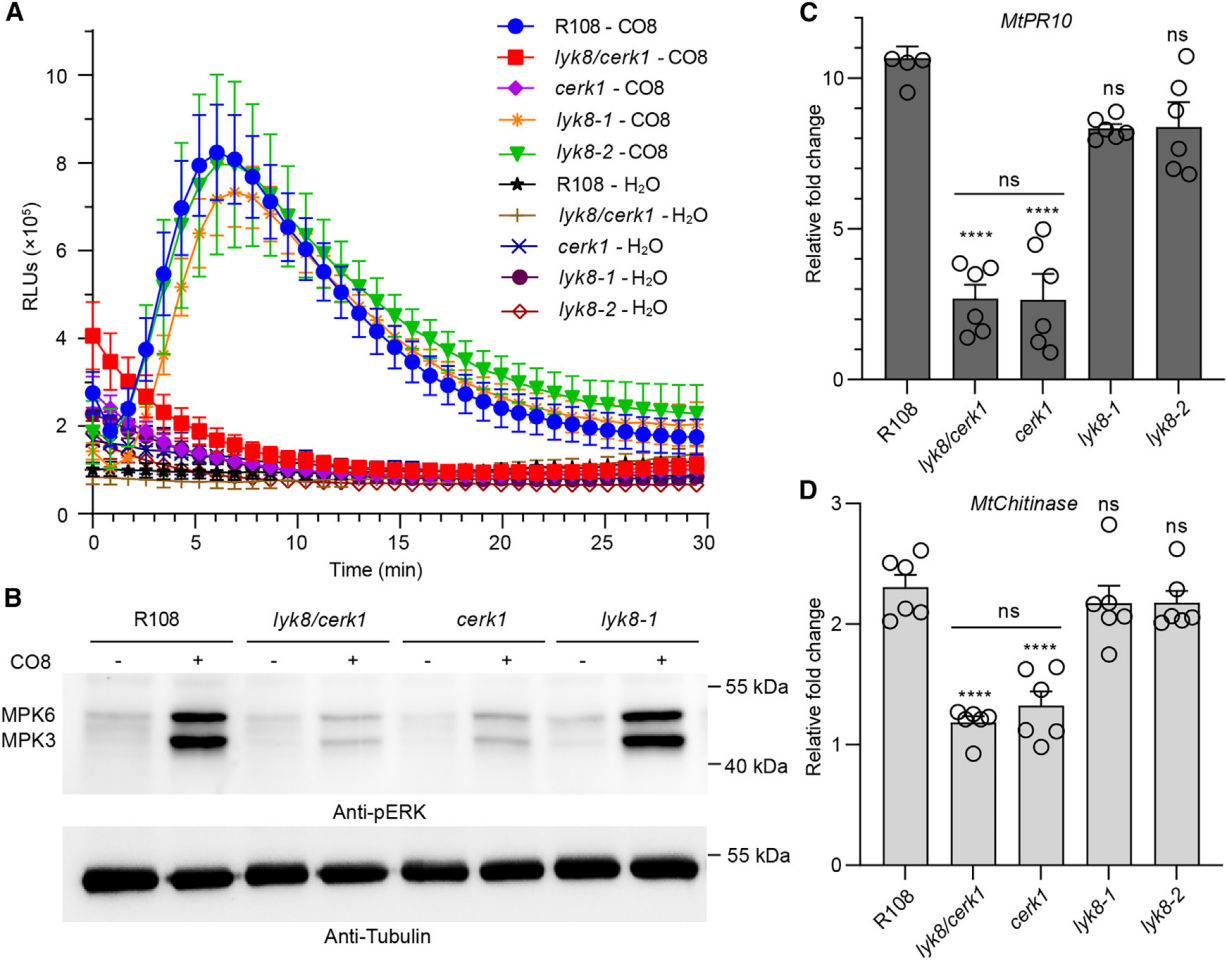
*LYK8* is not involved in CO8-triggered immune signaling (A) ROS production was measured in the roots of *M. truncatula* in response to both water and 10^−6^ M CO8 (mean ± SEM, n = 6). (B) The phosphorylation of MPK3 and MPK6 in plant roots was detected by the anti-pERK antibody. The Tubulin protein served as a control to ensure equal loading. (C and D) The real-time qPCR analysis of defense marker genes *MtPR10* (C) and *MtChitinase* (D) induced by 10^−6^ M CO8 in wild type and receptor mutants. The relative fold change compared with water treatment is shown. This experiment was independently replicated three times. The asterisks denote statistical significance as calculated by one-way ANOVA and Tukey’s multiple comparison (mean ± SEM, n = 8). See also Figure S6.

*CERK1* is implicated in both CO8-induced plant immunity and symbiosis.^7^ This prompted us to postulate a potential role for *LYK8* in plant immunity. To this end, we assessed early immune responses, specifically ROS production and MAPK activation, in the roots of wild type and receptor mutants following CO8 treatment. Notably, *lyk8* roots exhibited ROS response and MPK3/6 phosphorylation at levels comparable to those of the wild type (Figures 5A, 5B, S6A, and S6B). Although *cerk1* plants exhibited a modest activation of MPKs, this activation was *LYK8* independent, as shown by the similar levels of activation between *lyk8/cerk1* and *cerk1* (Figures 5B and S6B). This suggests no functional overlap between *LYK8* and *CERK1* in CO8-triggered MAPK activation. Furthermore, through real-time qPCR analysis, we examined CO8-induced expression of defense genes such as *MtPR10*, *MtChitinase*, and *MtRbohA*.^7^ *LYK8* is dispensable for the induction of these genes upon CO8 exposure and does not have a redundant role with *CERK1* in defense regulation (Figures 4C, 4D, and S6C). Consequently, these data indicate that LYK8 does not act as a redundant receptor alongside CERK1 in CO8-trigged immune signaling.

**Figure 6:**
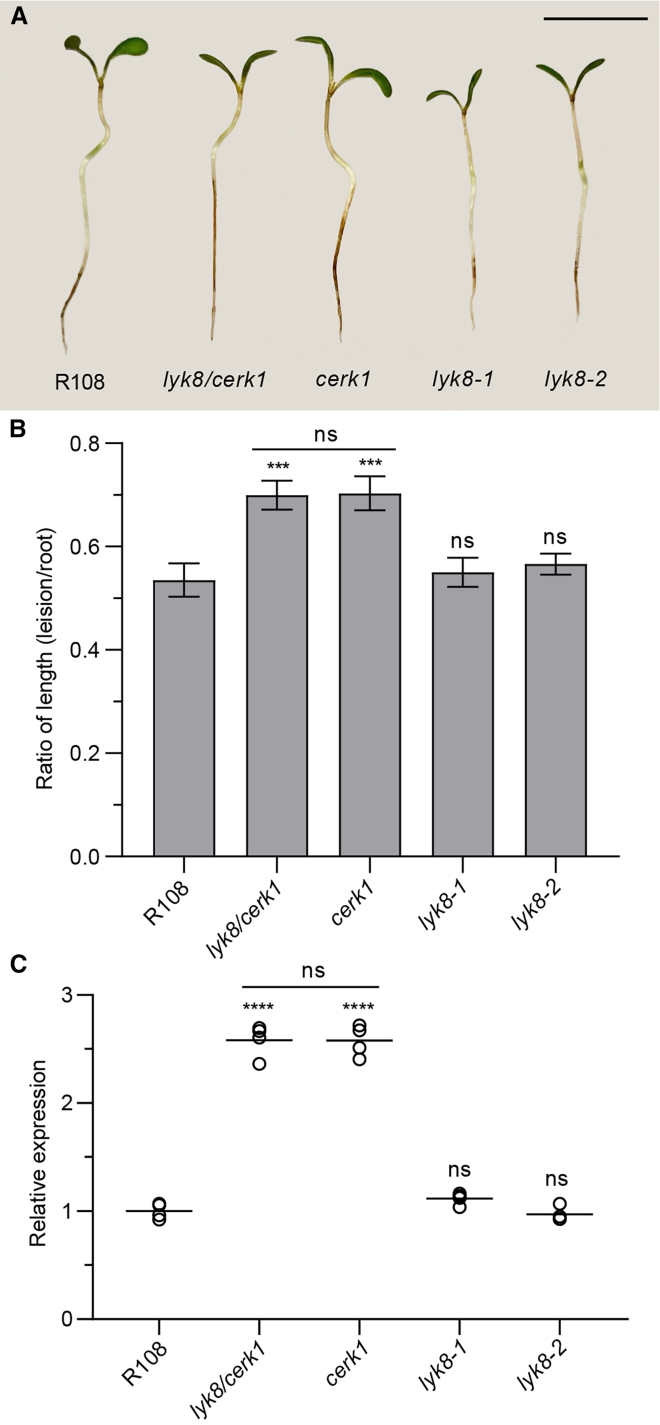
*LYK8* is dispensable for *M. truncatula* resistance against a fungal pathogen (A–C) 5-day-old *M. truncatula* seedling roots were inoculated with *F. oxysporum* spores. (A) Root disease symptoms (scale bars, 1 cm). (B) Quantification of the ratio of lesion size to the total length of the roots. (C) Assessment of *F. oxysporum* biomass measured using the fungal gene *FOW1*, relative to the *M. truncatula* gene *Ubiquitin*. (B and C) These experiments were independently replicated twice. Asterisks denote statistical significance as calculated by one-way ANOVA and Tukey’s multiple comparison (mean ± SEM, n = 15).

To investigate the role of *LYK8* in regulating plant disease resistance, we evaluated the susceptibility to the root fungal pathogen, *Fusarium oxysporum* (*F. oxysporum*), in both the wild type and receptor mutants. By measuring the root lesion size of the infected samples, we found that roots of *cerk1* were more susceptible to *F. oxysporum* (Figures 6A and 6B), while *lyk8* showed susceptibility similar to the wild type, and *lyk8/cerk1* exhibited susceptibility comparable to *cerk1* (Figures 6A and 6B). This observation is corroborated by real-time qPCR analyses quantifying *F. oxysporum* biomass, which reflected the fungal infection trends observed in the measurement of root lesion sizes (Figure 6C). Collectively, these findings indicate that *LYK8* is not involved in plant immunity.

### LYK8 forms a receptor complex with CERK1 for the activation of symbiosis signaling

**Figure 7:**
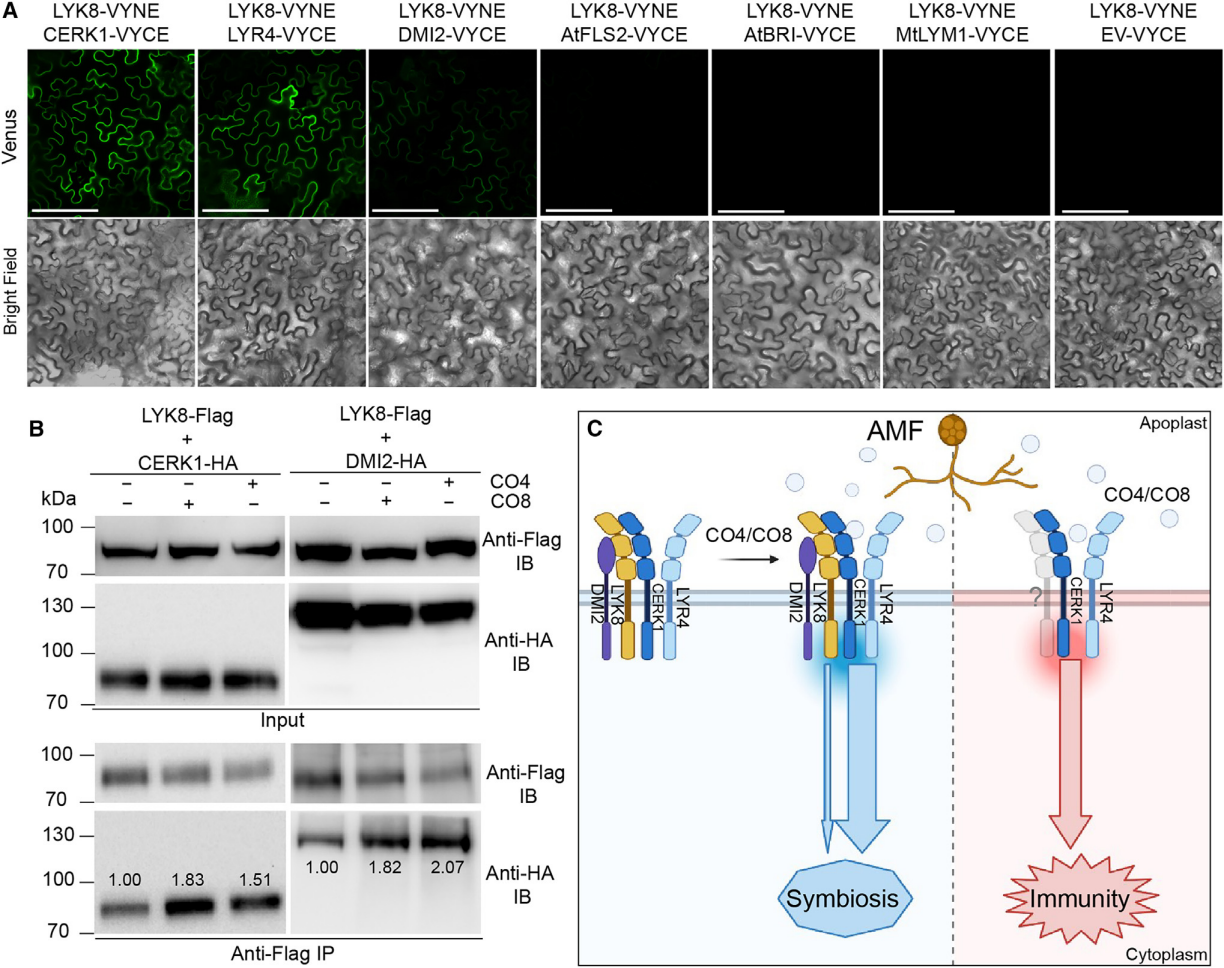
LYK8 interacts with CERK1 and DMI2, and these interactions are enhanced by CO4 and CO8 (A) LYK8 interacts with CERK1, LYR4, and DMI2, but not the plasma-membrane localized protein AtFLS2, AtBRI1, and MtLYM1 in *N. benthamiana* leaves, evidenced by split-Venus analysis. Scale bars, 150 μm. (B) CoIP validation of the interactions between LYK8 and CERK1/DMI2. The indicated proteins were transiently expressed in *N. benthamiana* leaves with or without treatment with 10^−6^ M of CO4 and CO8 for 10 min before protein extraction. The intensity of the bands for IP samples was quantified by ImageJ. (C) Proposed model of LYK8- and CERK1-mediated CO perception for activation of symbiosis and immunity. LYK8 is associated with CERK1/DMI2/LYR4 in the pre-symbiotic status. AMF-produced CO molecules, such as CO4 and CO8, stimulate LYK8 expression, strengthening its interaction with CERK1/DMI2 to initiate symbiosis signaling. CERK1, a key CO receptor, may partner with another LysM-RLK for immune signaling. See also Figure S7.

We hypothesized that LYK8 might partner with the identified CO receptors to form a receptor complex pivotal for the regulation of symbiosis signaling. To test this hypothesis, we employed a bimolecular fluorescence complementation (BiFC) assay, fusing LYK8 to the N-terminal part of yellow fluorescent protein Venus and the other receptors to the C-terminal part of Venus, subsequently expressing them in *N. benthamiana*. The results showed that LYK8 associates with CERK1 and LYR4 at the plasma membrane. However, we observed no interactions between LYK8 and the leucine-rich repeat receptor-like kinases AtFLS2^54^ and AtBRI1^55^ from *A. thaliana* or between LYK8 and the LysM receptor-like protein MtLYM1^56^ from *M. truncatula* (Figure 7A). Additionally, while LYK8 can interact with DMI2 on the membrane, the fluorescence appears weak due to low expression of DMI2 in *N. benthamiana* (Figure 7A). Extending our investigation using a co-immunoprecipitation assay, we confirmed interactions between LYK8 and other receptors, namely CERK1, LYR4, and DMI2 (Figure S7A). Upon exposure to CO4 and CO8, the interactions of LYK8 with CERK1 and DMI2 were notably enhanced, while the interactions of LYK8 with LYR4 or LYK8 themselves remained unchanged (Figures 7B and S7B). This implies that the CERK1-LYK8-DMI2 receptor complex might play a pivotal role in mediating CO-triggered symbiosis signaling.

## Discussion

AMF generate a range of signaling molecules, notably CO and LCO.^6^^,^^8^ These molecules activate symbiosis signaling and eventually enable host infection. This study reveals that CO-mediated symbiosis signaling is pivotal and sufficient to explain AMF root colonization, with *LYK8* working alongside *CERK1* for CO recognition (Figure 7C). The expression of *LYK8* is upregulated by CO, AMF, and nutrient deprivation^41^ (Figures S4A and S4B), implying a role of *LYK8* in reinforcing fungal perception, alongside constitutive *CERK1*. However, *CERK1*’s dominant role in CO signaling has overshadowed LYK8’s function, making it challenging to pinpoint mutants of LysM-RLKs that completely lack AMF colonization. The enhanced expression of *LYK8* and *CERK1* in arbuscule-containing cells suggests that CO recognition also occurs in the late stage of fungal infection. This is consistent with the observation of calcium oscillations in root cortical cells coincident with the fungal colonization of the root cortex,^57^ as well as the observation that application of CO can promote arbuscule development.^9^ This recognition likely promotes either the interactions between LYK8/CERK1 and CKL (CYCLIN-DEPENDENT-KINASE-LIKE) proteins or the phosphorylation of CKLs by the receptors, potentially enhancing lipid provisioning to the fungus.^58^ It seems that the role of *LYK8* in the AMS is evolutionarily conserved in dicots. For instance, in *P. andersonii*, concurrent mutations of the potential *LYK8*-orthologous gene, *PanLYK1*, and the *CERK1*-orthologous gene, *PanLYK3* (Figure S1G), lead to an absence of mature arbuscules in the cortical cells and a drastic reduction in fungal colonization.^20^ Similarly, suppressing the *LYK8*-ortholog *SlLYK12* in tomatoes severely inhibits the AMS.^59^ However, unlike *M. truncatula*, mutants in these receptors still partially sustain fungal colonization, implying the presence of other receptors or signaling pathways for the AMS in these species.

Beyond CO signaling, we previously showed that LCO signaling plays a role in the AMS,^7^ albeit to a lesser extent than CO signaling. AMF might leverage LCO molecules to amplify CO-mediated symbiosis signaling under specific scenarios. Supporting this, rice and barley roots respond to LCO only under nutrient-deprived conditions.^15^^,^^41^ Recent work indicates that LCO is not exclusive to AMF but is also synthesized by other beneficial and pathogenic fungi.^60^ This implies that the function of LCO extends beyond simply activating symbiosis, possibly acting as a general signal for fungi.^60^ This signal could either regulate the metabolic profile of microbes in the soil to compete across microbial kingdoms^61^ or influence various host responses, like enhancing lateral root growth and suppressing plant immunity.^7^^,^^8^^,^^15^^,^^46^^,^^48^^,^^51^^,^^62^^,^^63^^,^^64^^,^^65^^,^^66^^,^^67^ Importantly, our work demonstrates that abolishing CO signaling alone is sufficient to block AMF colonization, analogous to the situation observed in mutants of the symbiosis signaling pathway.^5^ As such, we conclude that CO recognition acts as the principal factor for recognizing AMF and is sufficient to explain the activation of symbiosis signaling by AMF. Consistent with our conclusion, the application of CO in plant roots has a long-term effect through the development of AMS signaling.^9^ We proposed that LCO must play much more subtle roles in AMF colonization, with their negative impact on immunity signaling^7^^,^^46^^,^^48^^,^^62^^,^^64^ being one modality of action.

The differential responses of *LYK8* and *CERK1* in mediating immunity are intriguing. *CERK1* functions in both immunity and symbiosis, while *LYK8* is dedicated to CO-mediated symbiosis signaling. It remains possible that *LYK8* might also contribute to immunity, potentially masked by the overlapping functions of other LysM-RLKs in *M. truncatula*. Current findings align with the observations on *SlLYK12* and *PanLYK1*, where both receptors appear to be important for AMF colonization, without direct roles in plant immunity.^20^^,^^59^ Additionally, the *L. japonicus* receptor *EPR3a*, which can bind β-1,3/β-1,6-glucans from fungi, appears to be involved only in symbiosis and is not required for β-glucan-triggered plant immunity.^68^ A recent study demonstrated that nanobody induction of the formation of a receptor complex can specifically activate symbiosis signaling.^69^ Interestingly, CO treatments intensify interactions of LYK8 with CERK1 and DMI2, but not LYR4 (Figures 7B and S7B), hinting that receptors might form specialized complexes in the presence of CO to differentiate between immunity and symbiosis (Figure 7C). The underlying mechanisms of these interactions remain an avenue for further study. Taken together, this suggests that plants might recognize identical molecules but employ different signaling routes for symbiosis and immunity, potentially due to the combination of receptors and their downstream associates.

LysM-RLKs, responsible for detecting CO and LCO, predominantly pair an active kinase from the *LYK* subfamily with an inactive one from the *LYR* subfamily.^11^ While *LYR4* is required for CO-induced symbiosis and immunity signaling, unlike *CERK1*, its mutation does not influence AMF colonization.^7^ This leads us to propose that other *LYR* receptors might be functionally interchangeable with *LYR4* for the AMS, much in the way *LYK8* is for *CERK1*. Notably, *cerk1* and *lyk8/cerk1* roots still maintain a similarly low level of MAPK activation in response to CO8 (Figure 5B). Considering *LYK8* is not involved in immunity signaling, this suggests that another *LYK* receptor might have functional redundancy with *CERK1*, specifically regulating immune signaling (Figure 7C). The expansion of the LysM-RLK family in legumes highlights the adaptive evolution of these receptors to engage in microbial associations. Future studies could focus on deciphering the functional diversity of LysM-RLKs and their downstream components in regulating plant immunity and symbiosis.

Our study extends the understanding of the molecular mechanisms underpinning the AMS. The newly discovered role of *LYK8*, in particular its synergy with *CERK1*, serves as an illuminating addition to the broader puzzle of how plants recognize AMF to engage in symbiosis. This knowledge could be pivotal in future efforts to optimize and harness AMS for agricultural advantage. In addition, our findings contribute to a deeper understanding of the complex interplay between CO-mediated symbiosis signaling and immunity, revealing new insights into the strategies plants employ to dissect the two different signaling pathways to interact with beneficial microbes while maintaining defense mechanisms.

## STAR★Methods

### Key resources table

**Table:** 

REAGENT or RESOURCE	SOURCE	IDENTIFIER
**Antibodies**		

Phospho-p44/42 MAPK (Erk1/2) (Thr202/Tyr204) Rabbit mAb	Cell Signaling Technology	Cat#4370; RRID: AB_2315112
Mouse monoclonal anti-Tubulin (clone B-5-1-2)	Sigma-Aldrich	Cat#T5168; RRID: AB_477579
Mouse monoclonal ANTI-FLAG M2-Peroxidase antibody	Sigma-Aldrich	Cat#A8592-2MG; RRID: AB_439702
Mouse monoclonal anti-GFP (clones 7.1 and 13.1)	Roche	Cat# 11814460001; RRID: AB_390913
Rat monoclonal Anti-HA-Peroxidase antibody	Roche	Cat# 12013819001; RRID: AB_390917
Goat anti-mouse IgG (H+L)-HRP Conjugate	Biorad	Cat# 1706516; RRID: AB_2921252
Goat anti-rabbit IgG-HRP Conjugate	Sigma-Aldrich	Cat# A0545-1ML; RRID: AB_257896

**Bacterial and fungal strains**		

*Ensifer meliloti Em*1021	Lab stock	N/A
*Agrobacterium tumefaciens* GV3101	Lab stock	N/A
*Agrobacterium rhizogenes* AR1193	Lab stock	N/A
*Fusarium oxysporum*	From Stephen Marek	N/A
*Rhizophagus irregularis*	Premier Tech	N/A
*Gigaspora margarita*	From Paola Bonfante	N/A

**Chemicals, peptides, and recombinant proteins**		

X-Gluc	Goldbio	B735
WGA-Alexa Fluor 488	Thermo Fisher	W11261
Aminoethoxyvinylglycine (AVG)	Sigma-Aldrich	A6685
CO4	Megazyme	O-CHI4
CO8	From Sébastien Fort	N/A
LCOs	From Sébastien Fort	N/A
CO5-biotin	Cullimore et al.^29^	N/A
CO7-biotin	Cullimore et al.^29^	N/A
Peroxidase from horseradish	Sigma-Aldrich	P6782
L-012	Wako chemicals	120-04891
cOmplete EDTA-free Protease Inhibitor Cocktail	Roche	11873580001
PhosSTOP	Roche	4906837001
Chitin Resin	New England Biolabs	S6651
ANTI-FLAG M2 Affinity Agarose Gel	Sigma-Aldrich	A2220
ChromoTek GFP-Trap® Magnetic Agarose	Proteintech	gtma
Streptavidin, HRP conjugate	Thermo Fisher	S911
RQ1 RNase-Free DNase	Promega	M6101
Typan Blue	Sigma-Aldrich	T6146

**Critical commercial assays**		

NEBridge® Ligase Master Mix	New England Biolabs	M1100S
BsaI-HF®v2	New England Biolabs	R3733
BbsI-HF	New England Biolabs	R3539
LunaScript RT SuperMix Kit	New England Biolabs	E3130
Luna Universal qPCR Master Mix	New England Biolabs	M3003
OneTaq Quick-Load 2X Master Mix	New England Biolabs	M0486
Spectrum™ Plant Total RNA Kit	Sigma-Aldrich	STRN250
Turface MVP	American Plant Products & Services	N/A
Vermiculite	American Plant Products & Services	N/A

**Experimental models: Organisms/strains**		

*M. truncatula R108*	Lab stock	N/A
*M. truncatula R108 YC3.6*	Feng et al.^7^	N/A
*M. truncatula cerk1*	Feng et al.^7^	NF16753
*M. truncatula nfp-3*	Feng et al.^7^	NF7796
*M. truncatula lyk6*	Oklahoma State University	NF14155
*M. truncatula lyk7*	Oklahoma State University	NF8175
*M. truncatula lyk8-1*	Oklahoma State University	NF9395
*M. truncatula lyk8-2*	Oklahoma State University	NF11260
*M. truncatula lyk10*	Oklahoma State University	NF20763
*M. truncatula lyk8/cerk1*	This study	N/A
*M. truncatula lyk8/nfp*	This study	N/A
*M. truncatula cerk1 YC3.6*	This study	N/A
*M. truncatula lyk8-1 YC3.6*	This study	N/A
*M. truncatula lyk8/cerk1 YC3.6*	This study	N/A
*M. truncatula lyk6/cerk1*	This study	N/A
*M. truncatula lyk7/cerk1*	This study	N/A
*M. truncatula lyk10/cerk1*	This study	N/A
*N. Benthamiana*	Lab stock	N/A

**Oligonucleotides**		

Primers	Table S1	N/A

**Recombinant DNA**		

pL2B-tYFPNLS-CERK1-3×FLAG-LYK8-3×HA	This study	N/A
pL2B-LAP1-proLYK8-GUS	This study	N/A
pL2B-LAP1-proCERK1-GUS	This study	N/A
pL1M-R2-pro35S-LYK8-3×HA-T35S	This study	N/A
pL1M-R2-pro35S-LYK8-3×FLAG-T35S	This study	N/A
pL1M-R2-pro35S-LYR4-3×HA-T35S	This study	N/A
pL1M-R2-pro35S-LYK3-3×HA-T35S	This study	N/A
pL1M-R2-pro35S-CERK1-3×HA-T35S	This study	N/A
pL1M-R1-pro35S-LYK8-VYNE-T35S	This study	N/A
pL1M-R2-pro35S-CERK1-VYCE-TOcs	This study	N/A
pL1M-R2-proLjUBI1-DMI2-VYCE-TOcs	This study	N/A
pL1M-R2-pro35S-LYR4-VYCE-TOcs	This study	N/A
pL1M-R2-pro35S-AtFLS2-VYCE-TOcs	This study	N/A
pL1M-R2-pro35S-AtBRI1-VYCE-TOcs	This study	N/A
pL1M-R2-pro35S-MtLYM1-VYCE-TOcs	This study	N/A
pL1M-R2-pro35S-VYCE-TOcs	This study	N/A
pL1M-R3-proLjUBI1-DMI2-3×HA-TNOS	This study	N/A
pL1M-R2-pro35S-AtFLS2-3×HA-T35S	This study	N/A
pL1M-R3-proLjUBI1-AtBRI1-3×HA-TNOS	This study	N/A
pL1M-R2-pro35S-MtLYM1-3×HA-T35S	This study	N/A
pCAMBIA-pro35S-LYK8ect-NFPtmic-YFP	This study	N/A
pCAMBIA-pro35S-MtLYR8ect-NFPtmic-YFP	Ding et al.^52^	N/A
pCAMBIA-pro35S-OsCEBiP8ect-NFPtmic-YFP	Cullimore et al.^29^	N/A

**Software and algorithms**		

ImageJ	National Institute of Health	Version 1.54f
ZEN Blue Version	Zeiss	Version 3.8
Prism	GraphPad Software	Version 9.5.0
MEGA	www.megasoftware.net/	Version 11
iTOL	itol.embl.de/	Version 6.8.1

### Resource availability

#### Lead contact

Further information and requests for resources and reagents should be directed to and will be fulfilled by the lead contact, Feng Feng (feng.feng@okstate.edu).

#### Materials availability

The plasmids and plant materials generated in this study can be provided upon request and following the requisite material transfer agreement (MTA) with the originating institution(s) responsible for generating the mutant line(s).

#### Data and code availability

• No sequencing data or accession numbers were produced as part of this study. Original data reported in this paper will be shared by the lead contact upon request.
• This paper does not report original code.
• Any additional information required to reanalyze the data reported in this paper is available from the lead contact upon request.

### Experimental model and subject details

#### Plant Materials and growth conditions

*M. truncatula* cv. R108 was used as the wild-type, *cerk1* (NF16753) and *nfp-3* (NF7796) mutants were reported previously.^7^ The *Tnt1* transposon insertion mutants *lyk6* (NF14155), *lyk7* (NF8175), *lyk10* (NF20763) *lyk8-1* (NF9395) and *lyk8-2* (NF11260) were obtained from the Oklahoma State University. The calcium reporter Yellow Cameleon (YC) 3.6 was introduced into the above-mentioned mutant lines by crossing them with an existing stable YC3.6 transgenic line. *M. truncatula* seeds were lightly scarified using sandpaper, sterilized in 10% sodium hypochlorite for 1.5 minutes, and then rinsed five times with sterilized water. The seeds were subsequently transferred to 1.5% water agar plates and stored in the dark at 4°C for three days before being germinated at room temperature. *S*eedlings were then either cultivated on responsive medium or grown in a mixture of soil substrates, depending on the requirements of the experiments (a 3:3:1 mixture of Turface MVP, play sand, and LC1 grower mix for mycorrhizal inoculation; a 1:1 mixture of vermiculite and Turface MVP for rhizobial inoculation). Five-week-old *Nicotiana benthamiana* leaves were used for protein expression. The plants were grown in a plant growth room at 22°C under a 16:8 photoperiod. Primers used for mutant identification are listed in Table S1.

#### Bacterial and Fungal Strains

*Ensifer meliloti* strain Em1021 was cultivated at 28°C on TY medium supplemented with appropriate antibiotics. *Agrobacterium tumefaciens* strain GV3101 and *Agrobacterium rhizogenes* strain AR1193 were incubated at 28°C on LB medium supplemented with antibiotics. *Rhizophagus irregularis* powder was purchased from Premier Tech, Canada or maintained in a carrot root organ culture as described previously.^51^ *Gigaspora margarita* BEG-34 was kindly provided by Professor Paola Bonfante and grown in co-culture with *Tagetes patula* (French marigold) in a sterile substrate of sand-terragreen mix in a glasshouse for approximately 4 months until maximum mycorrhizal colonization was achieved. The pots were then dried, plant material removed, and the substrate sieved to produce a crude inoculum containing spores and infective propagules. The inoculum was stored at 4°C under low (< 7 %) relative humidity. *Fusarium oxysporum* was originally isolated from alfalfa roots and cultivated on Synthetischer Nährstoffarmer Agar (SNA)^70^ for at least 7 days before collection of spores.

### METHOD DETAILS

#### Mycorrhizal inoculation

*M. truncatula* wild-type and mutant plants were cultivated in 48-well trays containing a mixture of soil substrates (3:3:1 mixture of Turface MVP, play sand, and LC1 grower mix). They were inoculated with *Rhizophagus irregularis* at a rate of 100 spores per plant for low-concentration inoculation and 300 spores per plant for high-concentration inoculation. For *Gigaspora margarita* inoculation, an inoculation strength of 5 % (v/v) per plant was used. For the co-cultivation of *lyk8/cerk1* plants with nurse plants, one wild-type *M. truncatula* R108 was grown alongside one *lyk8/cerk1* plant. Roots colonized by mycorrhizae were harvested at specified time points and subjected to staining with ink or 0.05% Trypan Blue. To assess the extent of mycorrhizal colonization, we used the gridline intersect method.^71^ Briefly, root segments were cut into smaller pieces and randomly distributed across a square Petri dish, which featured a 1 cm × 1 cm grid on its base. Using a Leica EZ4 stereomicroscope, 100 grid intersections were examined for each root sample to evaluate the AMF colonization and infection events. Representative images were taken using a Keyence VHX-5000 Digital Microscope (Keyence, UK).

#### Root architecture measurement

To quantify root development, root samples were harvested three weeks post-inoculation with *R. irregularis*, rinsed with tap water, and subsequently preserved in 50% (v/v) ethanol as described previously.^51^ The length of the primary root was determined using a ruler, and the first-order of lateral roots were counted using a Leica EZ4 stereomicroscope.

#### Nodulation test

Wild-type R108 and receptor mutant seedlings were grown in a 1:1 mixture of vermiculite and Turface MVP. The seedlings were watered twice weekly with B&D liquid medium and received additional tap water once per week. After 7 days of growth, the plant roots were inoculated with *E. meliloti* 1021 at an OD_600_ of 0.03. Following inoculation, the plants were continuously watered with B&D medium and tap water for three weeks. The roots were then harvested, rinsed with tap water, and the number of pink and white nodules on each root was quantified.

#### Molecular cloning and plant complementation

In the promoter-GUS assay, a 2.3 kb sequence upstream of either the *LYK8* or *CERK1* coding region was synthesized by Twist Bioscience, USA. This upstream sequence, serving as the native promoter, was then fused to the GUS reporter module to generate the construct by Golden Gate cloning,^72^ which was subsequently introduced into the *A. rhizogenes* AR1193 for hairy root transformation. To complement the *lyk8/cerk1* double mutant, we utilized Golden Gate cloning to create a single construct comprising both the *LYK8* and *CERK1* genes, as well as a nucleus localized triple YFP fluorescence marker (tYFP-NLS) for the selection of transformed plant roots. In this construct, the synthesized coding sequences of *LYK8* and *CERK1* were individually fused with a 3×HA and a 3×Flag peptide, respectively, each under the control of its own native promoter. We then introduced this construct into *A. rhizogenes* and transferred it into *lyk8/cerk1* plants through hairy root transformation. Roots with YFP signal and transformed with either an empty vector or with the *LYK8* and *CERK1* construct were then inoculated with *R. irregularis* spores. To assess the protein expression levels of LYK8 and CERK1, an equal amount of transformed roots was harvested three weeks post-AMF inoculation for protein extraction. Western blotting, employing anti-HA and anti-FLAG antibodies, was then conducted to verify the expression of LYK8 and CERK1 proteins in the complementation plants.

To generate constructs for protein expression in *N. benthamiana*, synthesized coding sequences of various genes, including *LYK8*, *CERK1*, *DMI2*, *LYR4*, *LYK3*, *AtFLS2*, *AtBRI1*, and *MtLYM1*, were fused with either a 3×HA or 3×FLAG tag. This resulted in the generation of constructs such as LYK8-HA, CERK1-HA, DMI2-HA, LYR4-HA, LYK3-HA, AtFLS2-HA, AtBRI1-HA, and MtLYM1-HA, as well as LYK8-FLAG. For the CO5/CO7 binding assay, we amplified the sequence representing the extracellular domain of LYK8 (LYK8ect, amino acids 1-223) from *M. truncatula* cDNA using PCR (primer details in Table S1). This sequence was then fused in-frame with the coding regions for the transmembrane and intracellular domains of MtNFP (NFPtmic), followed by a YFP tag, all under the control of CaMV 35S promoter. This fusion construct of LYK8 was used to maintain consistency with the constructs of two positive controls, MtLYR8ect-NFPtmic-YFP and OsCEBIPect-NFPtmic-YFP, which were created similarly.^29^^,^^52^^,^^73^ For the bimolecular fluorescence complementation (BiFC) assay, the *LYK8* coding sequence was fused with the N-terminal fragment of the yellow fluorescent protein Venus (VYNE). The coding sequences for *CERK1*, *LYR4*, *DMI2*, *AtFLS2*, *AtBRI1*, and *MtLYM1* were fused with the C-terminal fragment of Venus (VYCE). All these constructs were subsequently transformed into *Agrobacterium* GV3101 for transient expression in the leaves of *N. benthamiana*.

#### GUS and WGA staining

*M. truncatula* wild-type roots harboring a promoter-GUS construct, introduced through hairy root transformation, were cultivated in a soil mixture inoculated with mycorrhizal spores. These plant roots were harvested at intervals of one week, two weeks, and three weeks after inoculation. Subsequently, the roots were rinsed and subjected to GUS staining using 1 mg/mL X-Gluc staining buffer, which consists of 100 mM NaP buffer, 10 mM EDTA, and 1 mM potassium ferricyanide. The samples were incubated at 37°C for 6 hours and then washed with 70% ethanol and cleared in a 20% KOH solution. A final staining step was carried out using WGA-Alexa Fluor 488 dye (Thermo Fisher), dissolved in PBS buffer at a concentration of 0.5 μg/mL, and the samples were incubated for an additional 6 hours. Images of the stained samples were captured using a fluorescence microscope (BX51, Olympus, Japan).

#### Gene expression analysis

*M. truncatula* wild-type and mutant plants were cultured on Buffered Nodulation Media (BNM) agar plates for seven days. Afterward, the plants were moved to liquid BNM with or without adding elicitors such as 10^-7^ M CO4 (Megazyme), 10^-7^ M CO8 (produced by Sébastien Fort) and 10^-8^ M LCO (LCO from *E. meliloti* 1021, produced by Sébastien Fort). To induce symbiotic gene expression, the plant roots were treated with CO or LCO for 6 hours. For the induction of immune marker genes, a 30-minute incubation of CO8 was utilized. We used eight roots for each treatment and conducted three biological replicates for each time point. Regarding gene expression triggered by AMF, roots infected for 3 and 5 weeks were washed and collected for RNA extraction. The root samples were frozen using liquid nitrogen, and total RNA was extracted using a plant RNA extraction kit (Sigma). Genomic DNA was eliminated by treating with RNase-free DNase (Sigma) according to the manufacturer’s instructions. The concentration of the produced total RNA was determined using the NanoDrop-1000 Spectrophotometer (Thermo Fisher).

For real-time qPCR analysis, 300 ng of total RNA from each sample was utilized for cDNA synthesis with a LunaScript RT SuperMix Kit (New England Biolabs). Gene expression was determined using a CFX96 touch real-time PCR system (Bio-Rad), with 10 ng of cDNA template amplified using a Luna-SYBR green PCR master mix (New England Biolabs). Expression data were analyzed using an *M. truncatula* endogenous *Ubiquitin* gene as a reference. The fold change in gene expression was calculated for samples treated with elicitors or AMF in comparison to those subjected to water treatment or no AMF inoculation. For the Semi-quantitative RT-PCR analysis in Figures S1B and S1C, 10 ng of synthesized cDNA was added into PCR reaction. The primers for detecting the expression of symbiotic genes (*MtVapyrin*, *MtLYK10*, *MtD27*, *MtPT4*), defense genes (*MtPR10*, *MtChitinase*, *MtRbohA*), and the receptors (*LYK8*, *MtLYK6*, *MtLYK7*) can be found in Table S1.

#### Nuclear calcium oscillation

To assess periodic calcium oscillation, seedlings of *M. truncatula* wild-type and different receptor mutants containing the YC3.6 reporter were cultured on BNM agar plates supplemented with 100 nM AVG until lateral roots emerged. Only plant roots exhibiting strong YFP fluorescence were selected to assess the calcium response upon exposure to CO and LCO at specified doses. Calcium spiking was measured using an inverted epifluorescence microscope (TE2000, Nikon, Japan). Analysis of the fluorescent signal and the calcium spike curve followed the methodology outlined in a previous study.^15^

#### Reactive oxygen species production

*M. truncatula* wild-type and mutant plants were grown on BNM agar plates supplemented with 100 nM AVG for four days. Following this, the primary roots, which were about 4 cm in length, were segmented into 0.5 cm strips and placed in a 96-well microplate containing 200 μL of water in each well. An overnight incubation was conducted to eliminate internal ROS generated during the process of root segmentation. After incubation, water was removed from each well and replaced with 100 μL of reaction buffer containing 10^-6^ M CO8, 10 μg/mL horseradish peroxidase and 0.5 mM L-012. Luminescence was recorded with a Synergy H1 microplate reader (BioTek, USA) at indicated time points with a 1000 ms integration time. For each genotype and treatment, at least six samples were used as biological replication.

#### MAPK activity assay

Roots of four-day-old *M. truncatula* plants, cultivated on BNM agar plates supplemented with 100 nM AVG, were cut into small segments and subsequently incubated in water for 4 hours. The root samples were then exposed to either 10^-6^ M CO8 for 10 minutes or were left untreated before being rapidly frozen in liquid nitrogen for protein extraction. Root proteins were then homogenized using an extraction buffer composed of 50 mM HEPES-KOH pH 7.5, 50 mM NaCl, 1 mM PMSF, 5% Glycerol, 1 mM NaF, 1 mM EDTA, 0.2% Triton X-100, 2 mM DTT, complete protease inhibitors and the PhoSTOP phosphatase inhibitor. For the assessment of MPK3 and MPK6 phosphorylation, an anti-pERK antibody was employed. A duplicate blot was used to detect *M. truncatula* Tubulin, serving as the endogenous reference for equal loading, utilizing an anti-tubulin antibody.

#### Pathogen infection

*F. oxysporum* was cultured on SNA solid medium for a minimum of seven days. Sterilized, prechilled water (between 5-10 mL) was added to each plate and allowed to stand for 1 hour to release spores. The spore concentration was quantified using a hemacytometer. *M. truncatula* wild -type and mutant seedlings were grown on 0.8 % agarose plates for 36 hours. The root tip regions were inoculated with *F. oxysporum* spores at a concentration of 1 × 10^6^ spores/mL. For assessing *F. oxysporum* growth, 15 seedlings were collected at 48 hours post-inoculation to measure lesion size, which was then normalized against individual root length. The same root samples were then frozen in liquid nitrogen to extract DNA for quantitative PCR analysis, which utilized the *F. oxysporum FOW1*^74^ gene in relation to the *M. truncatula Ubiquitin* gene.

#### *Agrobacterium*-mediated transient expression

The *A. tumefaciens* GV3101 carrying the specified binary vectors were cultivated in Luria-Bertani (LB) medium, supplemented with the appropriate antibiotics, at 28 °C overnight. Following this step, the bacterial cells were collected and resuspended in an infiltration buffer (10 mM MgCl_2_, 10 mM MES, pH 5.7, and 100 μM acetosyringone), followed by a 3-hour incubation at 28 °C. The optical density of the bacterial suspension was adjusted to a final concentration of OD_600_ = 0.5. This suspension was then combined with *A. tumefaciens* containing a P19 protein before being infiltrated into the fully expanded *N. benthamiana* leaves using a syringe without a needle. Subsequently, the plants were covered with a black plastic bag for 12 hours to facilitate *A. tumefaciens* infection. Infiltrated leaves were collected at either 24 or 36 hours, depending on the specific experimental requirements.

#### BiFC assay

The *N. benthamiana* leaves expressing split-Venus proteins were harvested 36 hours after *A. tumefaciens* infiltration. To visualize the fluorescent signal, leaf tissue near the infiltration site was employed for microscopic analysis under a laser scanning confocal microscope (LSM 980, Zeiss, Germany), utilizing YFP filters: 514 nm for excitation and 540 nm for emission.

#### Chitin beads and CO-biotin binding assays

*Agrobacterium* GV3101 transformants carrying the LYK8-HA, CERK1-HA, LYR4-HA, and LYK3-HA constructs were infiltrated into *N. benthamiana* leaves and left for 36 hours to induce protein expression. Subsequently, the infiltrated leaves were ground into fine powder using liquid nitrogen, and the total powder was mixed with 1 mL of cold lysis buffer (50 mM HEPES-KOH pH 7.5, 50 mM NaCl, 1 mM PMSF, 5% Glycerol, 1 mM NaF, 1 mM EDTA, 0.2% Triton X-100, 2 mM DTT, complete protease inhibitors, and 2% PVPP) for extracting receptor proteins. The protein lysate was then subjected to centrifugation, and the resulting supernatant was transferred to a new tube for the chitin binding assay. To initiate the binding process, 20 μL of chitin resin beads (New England Biolabs) were washed three times by adding 1 mL of lysis buffer (without PVPP) and subsequently incubated with the extracted proteins for 3 hours in a cold room. After incubation, the beads were centrifuged and washed once with 1 mL of wash buffer I (50 mM HEPES-KOH pH 7.5, 400 mM NaCl, 1 mM EDTA, 0.2% Triton X-100, and 2 mM DTT), followed by three washes with buffer II (buffer I without NaCl), and finally, a last wash with buffer III (buffer I without NaCl and Triton X-100). The bound protein was recovered by boiling beads with SDS loading buffer.

To assess the binding affinity of receptors with CO5 and CO7, constructs encoding LYK8ect-NFPtmic-YFP, MtLYR8ect-NFPtmic-YFP, and OsCEBIPect-NFPtmic-YFP were expressed in *N. benthamiana* leaves, and the membrane fractions were isolated as previously described.^39^ The synthesis of cross-linkable and biotinylated versions of CO5-biotin and CO7-biotin has been detailed in earlier work.^29^ Binding assays with different concentrations of CO-biotin were conducted using membrane fractions in a buffer (25 mM NaCacodylate pH 6.0, 1 mM MgCl_2_, 1 mM CaCl_2_, 250 mM Saccharose and protease inhibitors) for 1 hour on ice. After incubation, samples were centrifuged at 31,000 g for 30 minutes at 4 °C, and the pellets were resuspended in IP buffer (25 mM Tris-HCl pH 7.5, 150 mM NaCl, 10 % glycerol, supplemented with protease inhibitors and the phosphatase inhibitor). The proteins were then solubilized in the IP buffer containing 0.2 % DDM detergent for 1 hour at 4 °C and immunoprecipitated using GFP-trap magnetic agarose beads (ChromoTek). Following washing in IP buffer, proteins were eluted with Laemmli buffer. Western blot analysis utilized anti-HA, anti-GFP and Streptavidin to verify the presence of the receptor proteins and their binding with chitin and CO-biotin, respectively.

#### Co-IP assay

*N. benthamiana* leaves were infiltrated with *Agrobacterium* GV3101 transformants carrying designated constructs and incubated for 36 hours to facilitate protein expression. Afterward, the leaves were infiltrated with 1 μM of CO4, CO8, or water for 10 minutes, followed by protein extraction. One gram of *Agrobacterium*-infected leaf tissue was homogenized into a fine powder in liquid nitrogen using a mortar and pestle. Protein extraction was performed by adding cold lysis buffer (50 mM HEPES-KOH pH 7.5, 150 mM NaCl, 1 mM PMSF, 5% Glycerol, 1 mM NaF, 1 mM EDTA, 0.5 % Triton X-100, 2 mM DTT, complete protease inhibitors, and 2% PVPP) to the samples, followed by a 10-minute incubation on ice. Subsequently, the protein lysate underwent centrifugation, and the resulting supernatants were filtered through a 0.45 μm filter. Ten microliters of cell extracts were used as input to demonstrate the expression of each protein. The remaining cell extracts were combined with 25 μL of pre-washed anti-FLAG M2 agarose resin beads (Sigma) and incubated for 4 hours at 4 °C. Afterward, the agarose beads were subjected to centrifugation and washed with washing buffers as detailed in the chitin binding section. The protein complexes associated with the beads were recovered by boiling them with SDS loading buffer for 5 minutes. The presence of HA- and FLAG-tagged proteins was subsequently assessed using anti-HA and anti-FLAG antibodies in a western blot analysis.

#### Phylogenetic analysis

Protein sequences of the LYK-type LysM-RLK family across several species, including *M. truncatula*, *L. japonicus*, *S. lycopersicum*, *O.sativa*, *Brachypodium distachyon*, *A. thaliana* and *P. andersonii* were obtained from National Center for Biotechnology Information (details listed in Table S2). These sequences were aligned using MUSCLE. Subsequently, a neighbor-joining phylogenetic tree was constructed using MEGA software and modified by iTOL website.

### Quantification and statistical analysis

Statistical analysis was performed using GraphPad Prism 9.5.0 software. This analysis involved employing one-way or two-way analysis of variance (ANOVA) followed by Tukey’s multiple comparisons test or multiple Student’s *t*-tests. The error bars represent the standard error of the mean (S.E.M.). Statistically significant differences were denoted by asterisks (ns, p > 0.05; ^∗^, p < 0.05; ^∗∗^, p < 0.01; ^∗∗∗^, p < 0.001, ^∗∗∗∗^, p < 0.0001) to indicate samples with significant distinctions. The real-time qPCR data were shown using dot plots to reflect the data distribution, and the normality of samples was confirmed by the Shapiro-Wilk test. Detailed sample size and statistical differences are available in the figure legends.

## Supplemental information

Document S1. Figures S1–S7 and Tables S1 and S2

Document S2. Article plus supplemental information
