# Supplementary material for: A receptor required for chitin perception facilitates arbuscular mycorrhizal associations and distinguishes root symbiosis from immunity
Source: Curr Biol. 2024 Apr 22;34(8):1705–1717.e6. doi: 10.1016/j.cub.2024.03.015 (PMC11037463; doi:10.1016/j.cub.2024.03.015)
Supplement: Document S1. Figures S1–S7 and Tables S1 and S2 [file mmc1.pdf]

Current Biology, Volume *34*

Supplemental Information

A receptor required for chitin perception facilitates arbuscular mycorrhizal associations and distinguishes root symbiosis from immunity

Jingyi Zhang, Jongho Sun, Chai Hao Chiu, David Landry, Kangping Li, Jiangqi Wen, Kirankumar S. Mysore, Sébastien Fort, Benoit Lefebvre, Giles E.D. Oldroyd, and Feng Feng

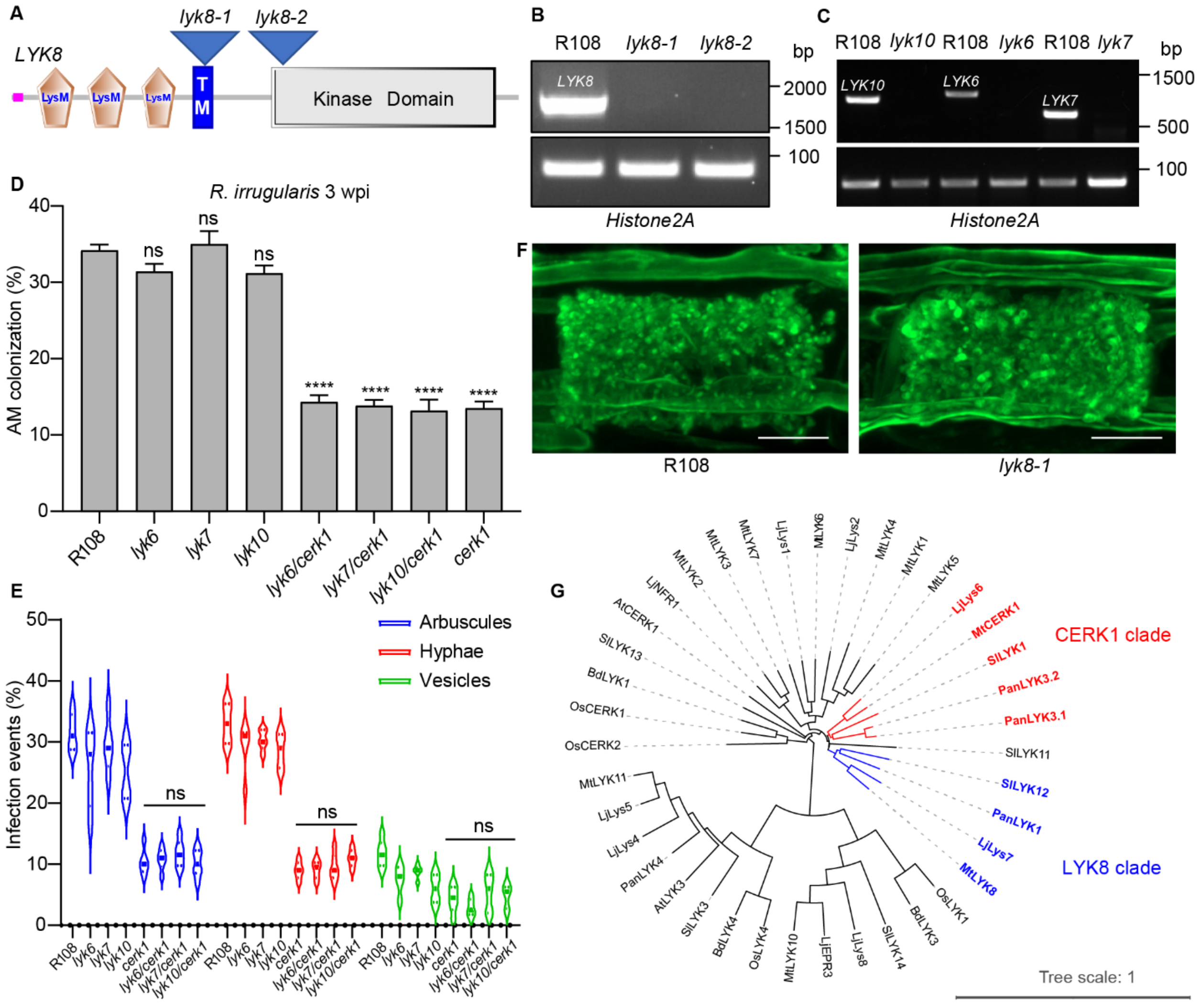

Figure S1. Genetic screen of single and double mutants of *M.truncatula* LysM-RLKs in AMF symbiosis. Related to Figure 1.

(A) The diagram illustrates the protein domains of LYK8 and highlights the positioning of *Tnt1* insertions associated with the mutant alleles. The LYK8 protein contains three extracellular LysM domains, a transmembrane domain (TM), and an intracellular kinase domain. Specifically, the *Tnt1* insertion situated on the TM domain is referred to as *lyk8-1*, whereas the insertion located on the kinase domain is identified as *lyk8-2*.

(B and C) Semi-quantitative RT-PCR analyses were conducted to assess the transcript levels of *LYK8*, *LYK6*, *LYK7*, and *LYK10* in both the wild-type and individual mutants of *M. truncatula*. *M. truncatula Histone 2A* was used as a loading control.

(D) Root colonization by *R. irregularis* was examined at 3-weeks post-inoculation (wpi) in both wild-type and various combinations of *cerk1* with distinct LysM receptor mutants. The extent of colonization is shown as a percentage of the total root length.
(E) Infection events were quantified within the roots of plants in panel (D).
(F) Confocal images of arbuscule structures in the wild-type and mutant. Bar = 10 μm.
(G) Phylogenetic analysis of LYK-type proteins. A maximum likelihood phylogenetic tree was constructed using protein sequences of all LYK proteins from *M. truncatula* (Mt), *L. japonicus* (Lj), *S. lycopersicum* (Sl), *O. sativa* (Os), *Brachypodium distachyon* (Bd), *A. thaliana* (At), and *P. andersonii* (Pan). The LYK8 clade is highlighted in blue, while the CERK1 clade is shown in red.
(D and E) These experiments were repeated three times with similar results. Asterisks denote statistical significance as calculated by one-way ANOVA and Tukey's multiple comparison. (Mean ± S.E.M., n = 10; ns, not significant).

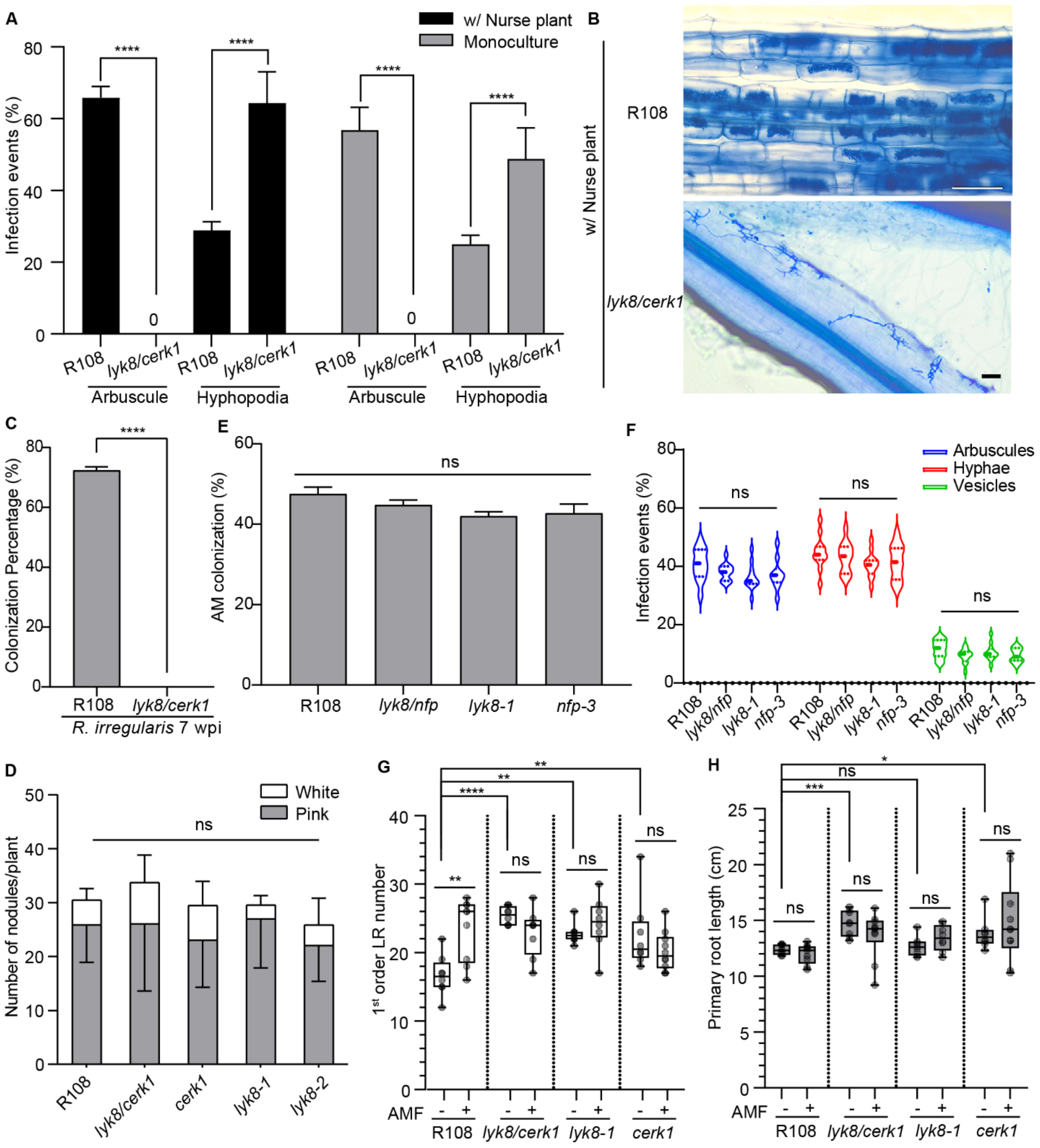

Figure S2. The phenotype of wild-type and receptor mutants in mycorrhization, nodulation, and AMF-induced lateral root development. Related to Figure 1.

(A) The roots of *lyk8/cerk1* plant lost the ability to form arbuscules even when co-cultured with wild-type plants. Arbuscules and hyphopodia were quantified in both wild-type and *lyk8/cerk1* mutants grown with or without wild-type nurse plants during the late infection stage (six weeks

after *R. irregularis* inoculation). Statistically significant differences were determined using Student's *t*-test. (Mean ± S.E.M., n = 10).

(B) Images displaying ink-stained colonization of AMF in the roots of *M. truncatula* wild-type and mutant plants cultivated alongside nurse plants. Bar=100 μm.

(C) Colonization of *R. irregularis* in wild-type and *lyk8/cerk1* mutant at 7 wpi.

(D) The nodulation phenotype of *M. truncatula* wild-type and various mutants was assessed upon inoculation with *E. meliloti* 1021. The count of white and pink nodules for each genotype was evaluated at three-weeks post-inoculation. Statistical significance was established through one-way ANOVA followed by Tukey's multiple comparison test. (Mean ± S.E.M., n = 15; ns, not significant).

(E) The degree of colonization by *R. irregularis* was evaluated as a percentage of root length in wild-type R108, *lyk8-1*, *nfp-3*, and *lyk8/nfp* mutant plants at 3 wpi.

(F) The Violin plot illustrates different infection events in the plant roots as depicted above.

(G and H) The number of first-order lateral roots (G) and the length of the primary root (H) were measured in wild-type plants and *lyk8/cerk1*, *lyk8*, and *cerk1* mutants three weeks after inoculation with *R. irregularis*. Statistically significant differences between groups are determined by Student's *t*-test. (Mean ± S.E.M., n = 8).

(D, E and F) These experiments were repeated three times with similar results. Asterisks denote statistical significance as calculated by one-way ANOVA and Tukey's multiple comparison. (Mean ± S.E.M., n = 10; ns, not significant).

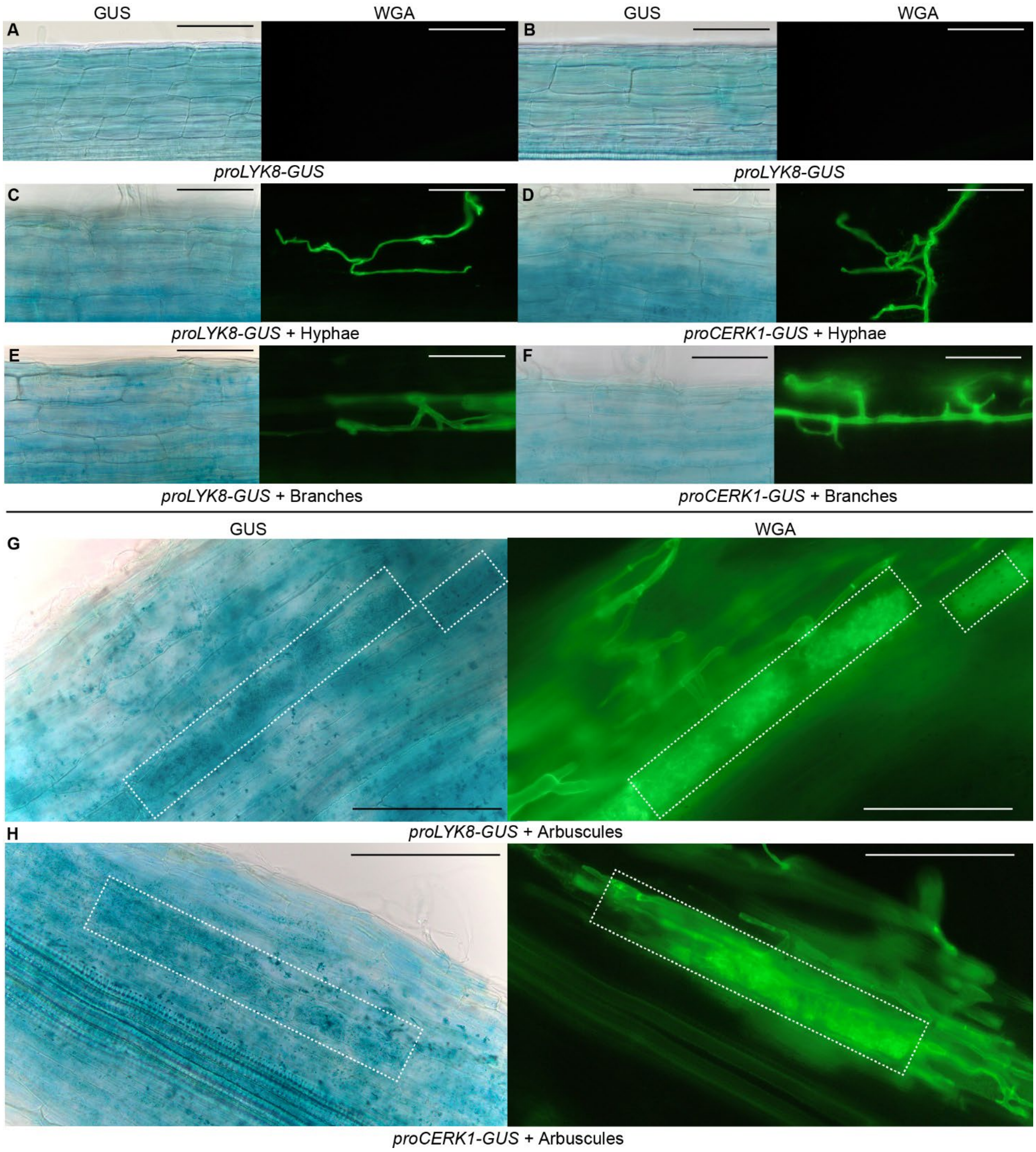

Figure S3. The expression of *LYK8* and *CERK1* was slightly enhanced in arbuscule-containing cells at late stage of AMF infection. Related to Figure 1.

(A-H) GUS and WGA staining were performed on the roots of *M. truncatula* wild-type plants transformed with promoter-GUS constructs to illustrate the expression patterns of *LYK8* and *CERK1* in the presence or absence of AMF. Bar = 150 μm.

(A and B) The GUS activity of *LYK8* and *CERK1* promoters in the roots, in the absence of AMF, showed a ubiquitous expression pattern across all root cell layers.

(C-F) GUS activity was detected at the early stage of AMF infection, associated with hyphopodia formation (C and D), and hyphal branching inside root cells (E and F).

(G and H) GUS activity exhibited a slight enhancement in cells containing arbuscules, which are marked with white dashed rectangles.

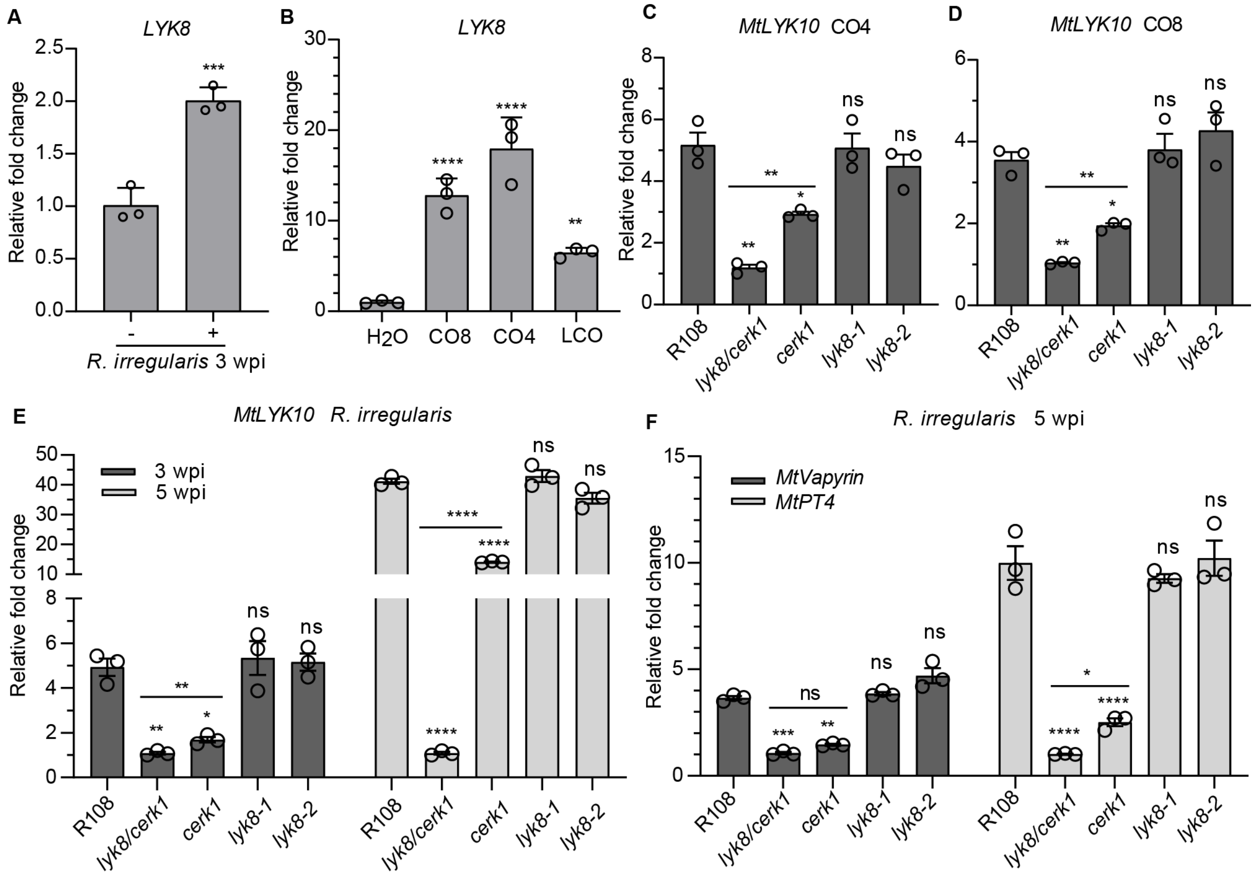

Figure S4. *LYK8* can be induced by COs, LCO and AMF, and it regulates the expression of symbiotic genes in conjunction with *CERK1*. Related to Figure 2.

(A) and (B) qRT-PCR analysis was conducted to assess the expression of *LYK8* in the roots of *M. truncatula* wild-type plants after treatments with *R. irregularis* (A) or 10^{-8} M of CO4, CO8 and LCO (LCO produced by *E. meliloti* 1021) (B). Statistically significant differences in (A) were determined using Student's *t*-test. (Mean ± S.E.M., n = 8).

(C-E) The qRT-PCR analysis examined the expression of the symbiotic marker gene, *MtLYK10,* in wild-type and different receptor mutants in response to CO4 (C), CO8 (D) treatment for 6 hours, as well as *R. irregularris* (E) treatment at 3 and 5 wpi.

(F) The expression of *MtVapyrin* and *MtD27* was evaluated in the roots of both wild-type and mutant plants following inoculation with *R. irregularis*.

(B-F) These results were based on three independent biological replicates. Asterisks denote statistical significance as calculated by one-way ANOVA and Tukey's multiple comparison. (Mean ± S.E.M., n = 8).

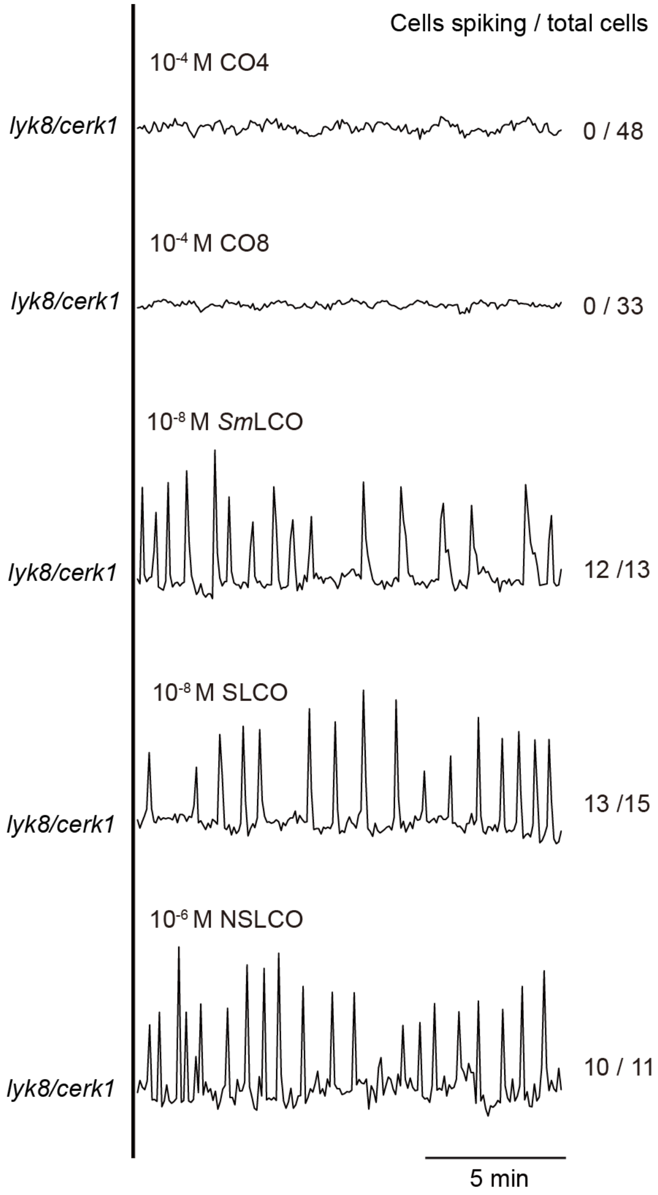

Figure S5. CO but not LCO-triggered calcium oscillations were completely abolished in *lyk8/cerk1* mutant. Related to Figure 3.

Representative calcium oscillations were captured in the atrichoblasts of lateral roots of *lyk8/cerk1*. These traces were recorded in roots responding to various concentrations of specific compounds: an exceptionally high concentration (10^{-4} M) of both CO4 and CO8, as well as more physiological concentrations of *Em*LCO (LCO produced by *E. meliloti* 1021, 10^{-8} M), S-LCO (sulfated LCO at 10^{-8} M), and NS-LCO (non-sulfated LCO at 10^{-6} M). These traces represent the ratio of YFP to CFP, expressed in arbitrary units. Annotations alongside each trace specify the count of responsive cells relative to the total number of cells assessed.

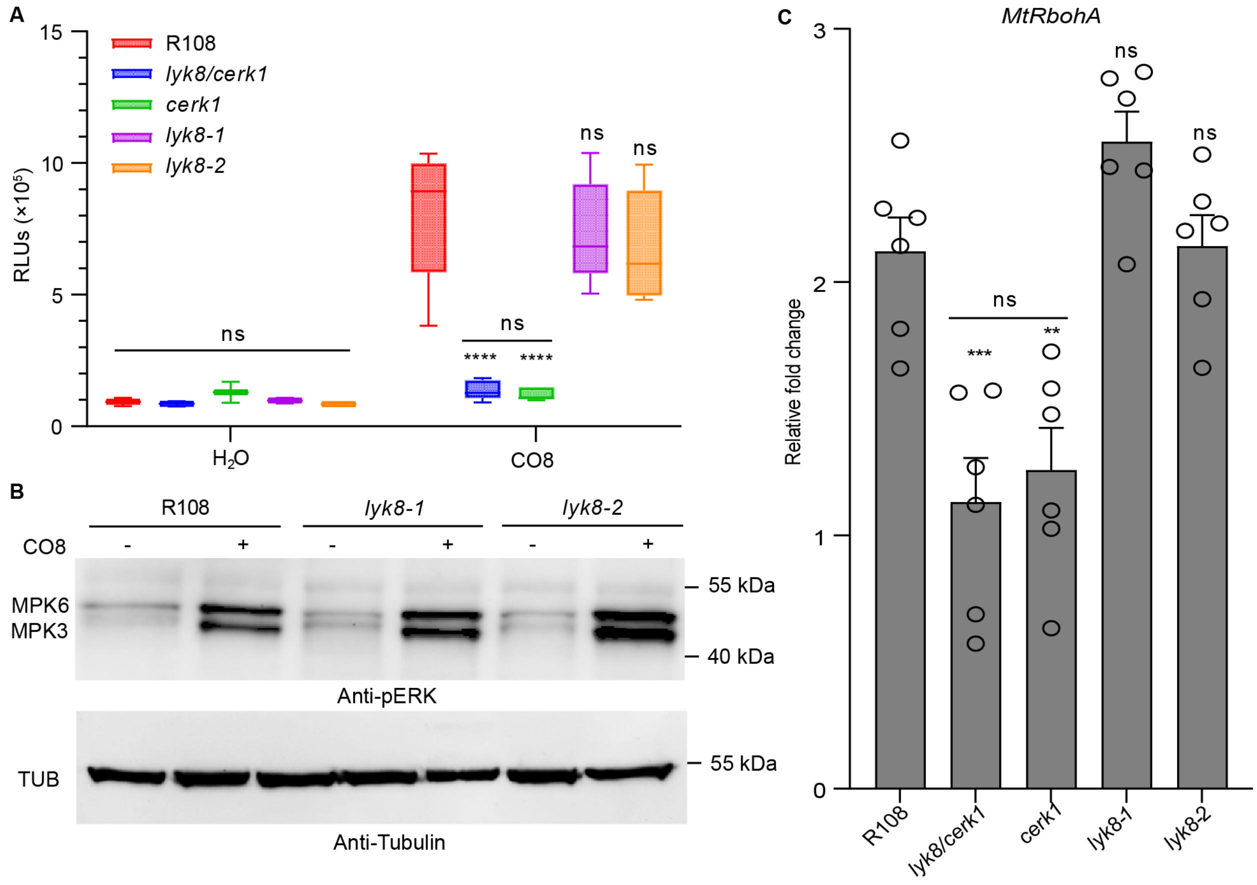

Figure S6. *LYK8* does not play a role in CO8-triggered immune signaling. Related to Figure 5.

(A) *M. truncatula* roots of wild-type R108, *lyk8-1*, *cerk1*, and *lyk8/cerk1* were treated with or without 10^{-6} M CO8 for ROS induction. ROS levels were quantified based on the average of the maximum relative luminescence units (RLUs) recorded in each curve from Figure 5A.

(B) MAPK activation in the roots of wild-type R108 as well as *lyk8-1* and *lyk8-2* mutants induced by 10^{-6} M CO8 for 10 minutes. The phosphorylation bands were detected using an anti-pERK antibody. *M. truncatula* Tubulin protein was used as the loading control.

(C) The qRT-PCR analysis of the expression levels of the defense marker gene *MtRbohA* in the roots of wild-type and receptor mutants following treatment with 10^{-7} M CO8.

(A and C) These experiments were independently replicated three times. Asterisks denote statistical significance as calculated by one-way ANOVA and Tukey's multiple comparison. (Mean ± S.E.M., n = 8; ns, not significant).

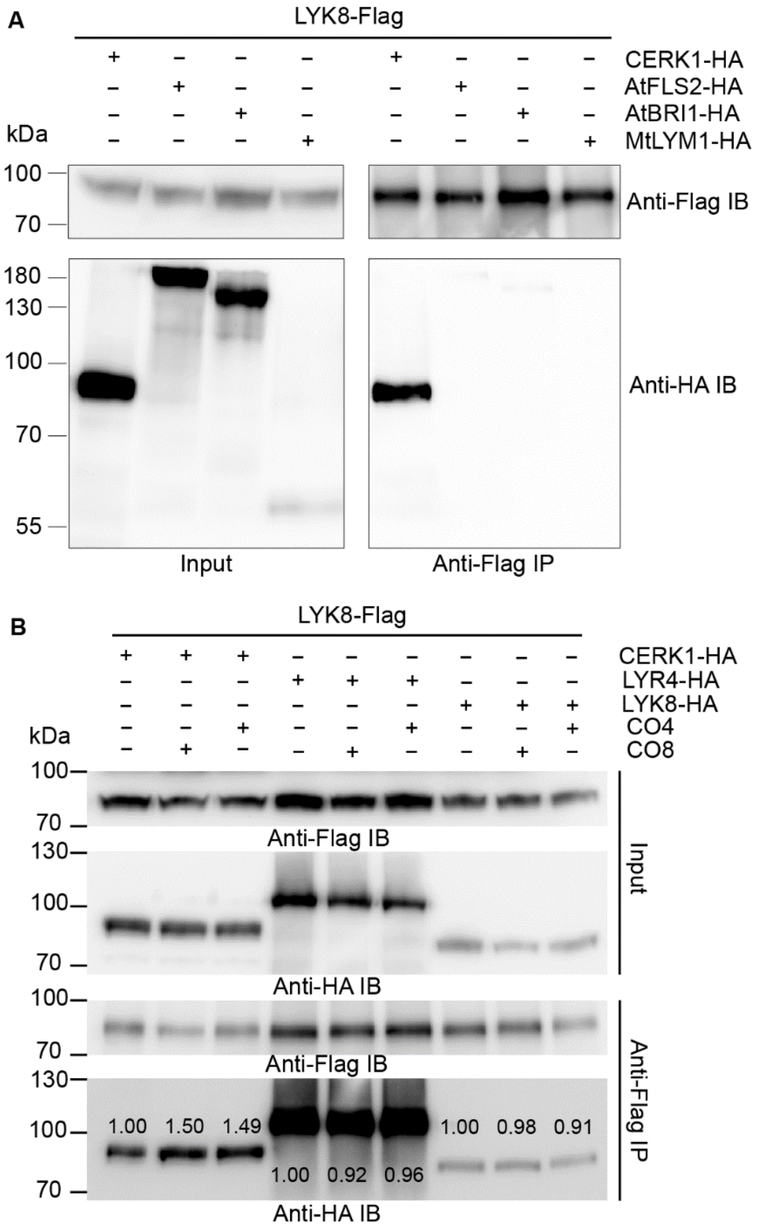

Figure S7. CO treatment can promote interactions between LYK8 and CERK1, but not LYR4 and LYK8 itself. Related to Figure 7.

(A and B) The indicated constructs were transiently expressed in *N. benthamiana* leaves, followed by Co-IP assays.

(A) LYK8 can specifically interact with CERK1 but not with other plasma membrane-localized proteins.

(B) LYK8 can interact with LYR4 and LYK8 independently of CO treatment. These interactions were not changed by pretreatment with 10^{-6} M CO4 or CO8. The intensity of the bands for IP samples was quantified by ImageJ.

Gene	Gene ID	Primer sequence (5' → 3')
**For mutants genotyping**		
lyk6	Medtr5g086040	F: GTCTTATCTTCTTTCTTACA<br>R: TGGCTGAAGCTCATAGAGAA
lyk7	Medtr5g086030	F: CATCTAAATTAATCGGAGTT<br>R: CAACTCAGCATCAATTTCAG
lyk8-1	Medtr2g024290	F: GCAGATGAAAATGGAAACTTTC<br>R: CAATTGTGTAAGTATTTAGGA
lyk8-2	Medtr2g024290	F: GGTATACTATGCCGAGCTACG<br>R: TGATGCAAGCAGCATTCTAAG
cerk1	Medtr3g080050	F: ATGGAACATCAACCCAGATTCACCT<br>R: CTTTTCCCGGAACAAAAACAATGC
lyk10	Medtr5g033490	F: GTCACCACTTTACCATGGAAAAG<br>R: CACAGATTCTGGTGGAAGGTAG
nfp	Medtr5g019040	F: ATGTCTGCCTTCTTTCTTCCTTC<br>R: ACGAGCTATTACAGAAGTAACAAC
Tnt1	N/A	F: TCCTTGTTGGATTGGTAGCC<br>R: CAGTGAACGAGCAGAACCTGTG
**For qPCR analysis**		
LYK8	Medtr2g024290	F: TACCAGGAGCATCTGAACT<br>R: TACCTGTCAACCTTGGAGAAAC
MtLYK10	Medtr5g033490	F: AGAAGCTACGAGCCAAGGTAGC<br>R: AGGTAGCCTGGTGTTCCAACAAG
MtRbohA	Medtr1g083290	F: TTCGAACTTTGGGCGATTGGAC<br>R: ATTCGTAGCCTTGGCATCCTTGG
MtPR10	Medtr5g033490	F: GGCTCAAATGGAGGGTCTATTG<br>R: GCTTTGCCTTCCTCAACCT
MtChitinase	Medtr2g099470	F: GGCTGACATCCTTACACAAGA<br>R: AGAATTGAGGGCATCGAGAAA
MtVapyrin	Medtr6g027840	F: GCCAGTTGCATTTAGGATTCA<br>R: GCACCTGGAGCAAGAACACT
MtD27	Medtr1g471050	F: AGTTCTTGCAAGGCCTACAGATG<br>R: TGATTCCTGTTGCTGCTTGAACAC
MtPT4	Medtr1g028600	F: GACACGAGGCGCTTTCATAGCAGC<br>R: GTCATCGCAGCTGGAACAGCACCG
MtUbiquitin	Medtr4g088485	F: AACTTGTTGCATGGGTCTTGA<br>R: CATTAAGTTTGACAAAGAGAAAGAGACAGA
FOW1	AB078975	F: GGTATCCTTGGTGGTGTCTCC<br>R: CTACCCCAGTTGGTCATCAGT
**For plasmid construction**		
pro35s-LYK8ect-NFPtmic-YFP		F: GGTCTCACAAAATGATCACAAATCAAATTTTCA<br>R: GGTCTCGTAAGGCCTCCTTTGGACATTCC

Table S1. Primers used in this work. Related to STAR Methods

Protein	Species	NCBI accession number
LYK1	*Medicago truncatula* (Mt)	AAQ73154
LYK2		CAN88845
LYK3		Q6UD73
LYK4		AES99915
LYK5		AES99912
LYK6		Q6UD75
LYK7		AAQ73158
LYK8		XP_013462891
CERK1		XP_003601376
LYK10		XP_003613165
LYK11		XP_003627045
NFR1	*Lotus japonicus* (Lj)	CAE02590
Lys1		BAI79267
Lys2		BAI79268
EPR3		BAI79269
Lys4		BAI79271
Lys5		BAI79272
CERK6		BAI79273
Lys7		BAI79274
Lys8		Not registered,<br>Gene ID: LotjaGi4g1v0157000.1
LYK1	*Solanum lycopersicum* (Sl)	NP_001233773
LYK3		XP_010318399
LYK11		NP_001234719
LYK12		NP_001234725
LYK13		NP_001234730
LYK14		XP_019069864
LYK1	*Oryza sativa* (Os)	XP_015628733
LYK4		XP_015633426
CERK1		XP_015650771
CERK2		BAJ09794
LYK1	*Brachypodium distachyon* (Bd)	XP_010235348
LYK3		XP_010233416
LYK4		XP_003567102
CERK1	*Arabidopsis thaliana* (At)	NP_566689
LYK3		NP_175606
LYK1	*Parasponia andersonii* (Pan)	PON52141.1
LYK3.1		PON42545.1
LYK3.2		PON42546.1
LYK4		PON54359.1

Table S2. Protein IDs used in the phylogenetic study. Related to STAR Methods
